# Synthesis and antiplasmodial activity of regioisomers and epimers of second-generation dual acting ivermectin hybrids

**DOI:** 10.1038/s41598-021-04532-w

**Published:** 2022-01-12

**Authors:** Lovepreet Singh, Diana Fontinha, Denise Francisco, Miguel Prudêncio, Kamaljit Singh

**Affiliations:** 1 Department of Chemistry, Centre for Advanced Study, Guru Nanak Dev University, Amritsar, 143005 India; 2 Faculdade de Medicina da Universidade de Lisboa, Instituto de Medicina Molecular, Av. Prof. Egas Moniz, 1649-028 Lisbon, Portugal

**Keywords:** Structure-based drug design, Synthetic chemistry methodology

## Abstract

With its strong effect on vector-borne diseases, and insecticidal effect on mosquito vectors of malaria, inhibition of sporogonic and blood-stage development of *Plasmodium falciparum*, as well as in vitro and in vivo impairment of the *P. berghei* development inside hepatocytes, ivermectin (IVM) continues to represent an antimalarial therapeutic worthy of investigation. The in vitro activity of the first-generation IVM hybrids synthesized by appending the IVM macrolide with heterocyclic and organometallic antimalarial pharmacophores, against the blood-stage and liver-stage infections by *Plasmodium* parasites prompted us to design second-generation molecular hybrids of IVM. Here, a structural modification of IVM to produce novel molecular hybrids by using sub-structures of 4- and 8-aminoquinolines, the time-tested antiplasmodial agents used for treating the blood and hepatic stage of *Plasmodium* infections, respectively, is presented. Successful isolation of regioisomers and epimers has been demonstrated, and the evaluation of their in vitro antiplasmodial activity against both the blood stages of *P. falciparum* and the hepatic stages of *P. berghei* have been undertaken. These compounds displayed structure-dependent antiplasmodial activity, in the nM range, which was more potent than that of IVM, its aglycon or primaquine, highlighting the superiority of this hybridization strategy in designing new antiplasmodial agents.

## Introduction

Malaria is a protozoan infection caused by the parasites of the genus *Plasmodium*^1, 2^. While five species of *Plasmodium*, *P. falciparum, P. vivax, P. ovale, P. malariae,* and *P. knowlesi* are responsible for human malaria, severe and complicated malaria is mostly caused by *P. falciparum* and *P. vivax*^1–4^. Further, *P. falciparum* is accountable for most of the malaria-related deaths worldwide, estimated at about 409,000 deaths arising from approximately 229 million cases of malaria in 2019, as reported by the World Health Organization (WHO)^5^. Children under five years and pregnant women are among the most vulnerable group affected by malaria^1, 5–7^. An obligatory step of infection by malaria parasites starts with the clinically silent liver-stage infection, wherein *Plasmodium* sporozoites, delivered by female *Anopheles* mosquitoes invade hepatocytes and replicate inside a parasitophorous vacuole, inside the hepatic host cell. The latter provides protection as well as resources to the infectious parasite to multiply into thousands of daughter merozoites, which are eventually released to the bloodstream where they infect erythrocytes, leading to the onset of the symptomatic, cyclic blood stage of infection^1–4^. *P. vivax* infection tends to relapse even after successful treatment due to the persistence of uninucleate parasites known as hypnozoites, long-lived, dormant, hepatic parasite forms that can reactivate months or even years after initial infection^1, 8–10^. Malaria control efforts are additionally complicated by the coexistence of *P. vivax* with *P. falciparum* infection in endemic regions^11^.

**Figure 1:**
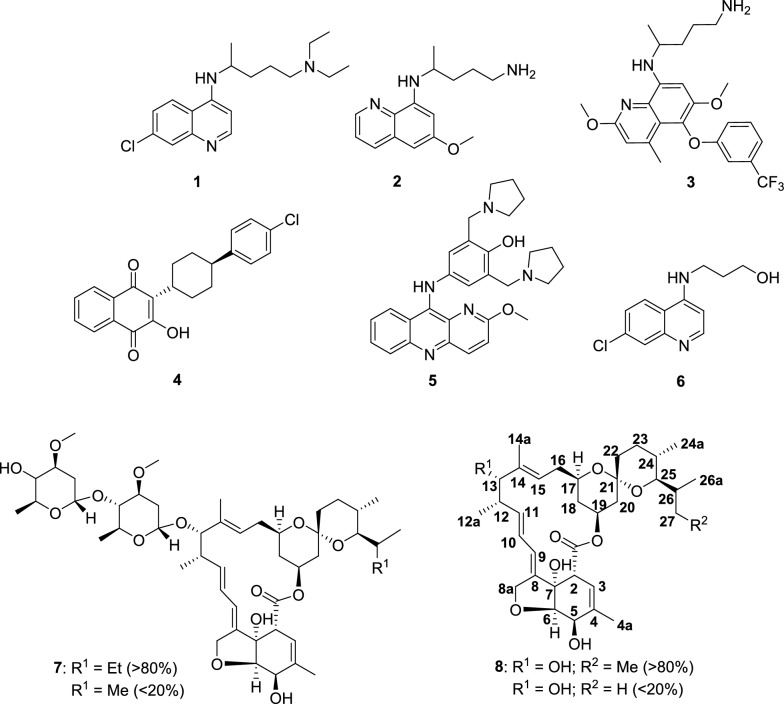
Structures of chloroquine **1**, primaquine **2**, tafenoquine **3**, atovaquone **4**, pyronaridine **5**, chloroquine analogue **6**, IVM **7**, IVM aglycon **8** and numbering scheme.

The key strategies used to manage malaria include preventive measures, such as insecticidal treated bed nets^12^, mosquito repellent indoor sprays^13^, and chemoprevention^1^, as well as therapeutic interventions, through the use of antimalarial medicines^14^. The most advanced malaria vaccine candidate, RTS, S/AS01(RTS,S), affords only partial protection, with efficacy as little as ∼34%, when tested on children living in *P. falciparum*-endemic areas, suggesting the need for the administration of multiple booster doses^15, 16^. Very recently, in October 2021, The WHO has recommended the use of RTS,S among children in sub-Saharan Africa and in regions with moderate to high *P. falciparum* malaria transmission^17^. However, this vaccine shows only moderate protection against severe disease caused by *P. falciparum*, while efforts to develop vaccines against *P. vivax* remain limited, despite the enormous burden caused by this parasite species^16, 18^. Traditional antimalarials based on chloroquine (**1**, CQ, Fig. 1) and 4-aminoquinolines^14^ are restricted by the development of drug resistance^19–21^, mainly due to over-use and single major mode of action i.e. inhibition of heme polymerization^19–22^. This leaves artemisinin-based combination therapies (ACTs)^14, 23^, representing fixed combinations of artemisinin or its analogs with a slow-acting antimalarial drug, as the most effective therapeutics for efficacious treatment of malaria^23, 24^. However, ACTs exclusively target the blood stage of infection, and only limited success has thus far been achieved in the development of drugs targeting the liver and transmission stages of the complex life cycle of malaria parasites^14, 23, 24^. It is thought that drug-resistant mutations arise in the sexual stages of the parasite’s life cycle where they are diploid and enter the asexual stages^19–21^. The 8-aminoquinolines, primaquine^25^ (**2**, PQ, Fig. 1) and tafenoquine **3**^26^, are the only drugs approved to eliminate *P*. *vivax* hypnozoites. However, these drugs face limitations of long dosage regimens, non-compliance with patients suffering from glucose-6-phosphate dehydrogenase deficiency (causes intravascular haemolysis, often requiring prescreening), and the fact that they are not recommended during pregnancy and lactation, restricting their widespread use^27^. Only two approved drugs, atovaquone (**4**, ATQ, Fig. 1) and pyronaridine (**5**, Fig. 1), have demonstrated multistage antiplasmodial activity^14, 28, 29^. Thus, finding new chemotherapeutic interventions that target the malaria parasite and/or the host, through the rational design of new drugs^30^ and/or repurposing of the time-tested drugs^31^, constitute one of the attractive strategies at the disposal of medicinal chemists.

Ivermectin (**7**, IVM, Fig. 1) (R^1^ = Et (B1a, > 80%); R^1^ = Me (B1b, < 20%), a semi-synthetic derivative of avermectin B_1_, is a powerful endectocide used in mass drug administration (MDA) against onchocerciasis^32^, lymphatic filariasis^33^, and several other parasitic diseases in humans^34–36^. The impact of IVM on malaria control is predominantly due to its insecticidal effect on the mosquito vectors carrying *Plasmodium* parasites^37, 38^, as well as to its inhibitory activity against mammalian and sporogonic^39^ parasite stages. IVM showed in vitro inhibitory effects against blood stages of *P. falciparum* infection (IC_50_ 1.56–2.85 μM) of both the chloroquine-resistant (CQ^R^) and chloroquine-sensitive (CQ^S^) strains of the parasite^40^, and suppressed parasitemia by 40% relative to placebo-treated controls in *P. berghei-*infected mice. In an independent study^41^, in vitro inhibitory effects of IVM against asexual and sexual stages of the *P. falciparum* (IC_50_ 100 nM and 500 nM, respectively) have been reported. However, in vivo studies revealed contradictory results, as no effect on parasitemia or gametocytemia was observed^41^. Further, IVM effectively reduces *P. berghei* infection of infected human hepatoma cells in vitro, with antiplasmodial activity comparable to the standard drug, **2** (IC_50_ = 2.1 and 2.4 μM, respectively). Additionally, IVM reduced liver infection in *P. berghei*-infected mice by 80% 44–46 h post IVM treatment^42^.

Further, IVM reduced in vitro development of *P. cynomolgy* schizonts (IC_50_ = 10.42 μM) and hypnozoites (IC_50_ = 29.24 μM) in rhesus macaque hepatocytes^43^. Finally, a recently-developed bioluminescence-based assay^44^ enabled demonstrating that IVM and other avermectins inhibit the sporogonic development of *P. berghei* in an in vitro setting^45^. Employing the rationale of hybrid drugs^46, 47^ by covalently linking of IVM sub unit with an antimalarial pharmacophore, “*first-generation*” molecular hybrids displayed structure-dependent “*dual-channel*” antiplasmodial activity in vitro against both the erythrocytic stages of *P. falciparum* and the hepatic stages of *P. berghei* infection^48^. However, these lacked a substantial insecticidal effect against *A. stephensi* mosquitoes in laboratory conditions, although, in a manner similar to IVM^48, 49^, it showed allosteric binding (in silico docking) to glutamate-gated chloride channels (GluCl) of the Cys-loop family, a primary target of IVM in *A. gambiae*^50^.

Inspired by these results^48^, we designed a set of “*second-generation”* IVM molecular hybrids using co-partners with established antiplasmodial activity and good pharmacokinetic properties. Herein, the first synthesis and improved antiplasmodial activity of the molecular hybrids of **7** in combination with CQ analogue **6** and **2**, linked through the primary hydroxyl^51^ and amine function, respectively to the C_13_-OH position of the IVM aglycon **8** is reported. We have also, for the first time isolated regioisomers and epimers of these hybrids (abbreviated as IVM-CQ and IVM-PQ) and tested their antiplasmodial activity. The “*second-generation*” hybrids are distinctly more active than their “*first-generation*” counterparts^48^, and demonstrate significant improvement of the “dual-channel” antiplasmodial activity. These results also provide a deeper understanding of the structure dependent antiplasmodial effects of molecular hybrids of **7** with other pharmacophores.

## Results and discussion

### Chemistry

#### Synthesis and isolation

**Figure 2:**
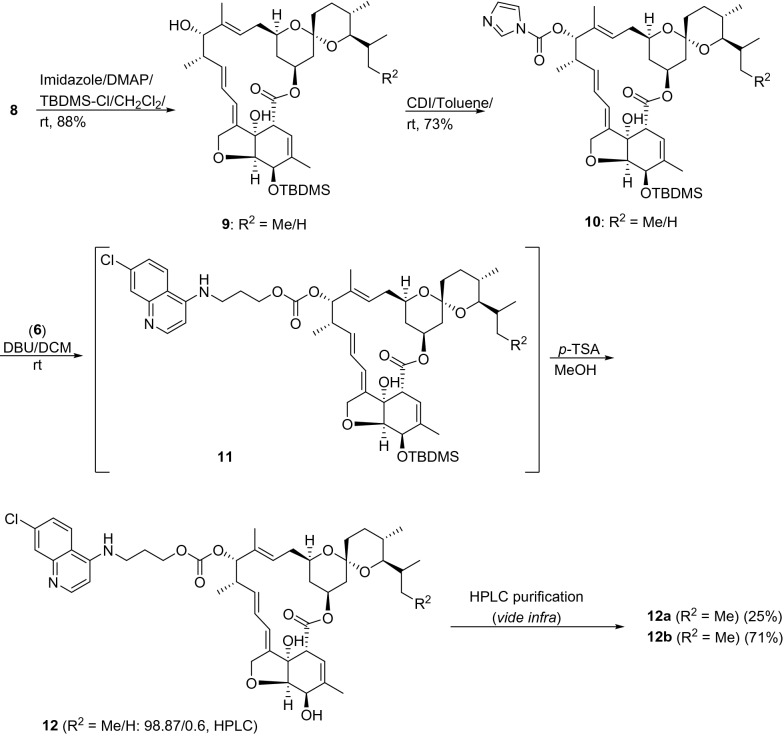
Synthesis of IVM-CQ conjugates **12** from **8**.

Routes for the synthesis of hybrids **12** and **15** from the common precursor **8** (Fig. 2) in combination with **6** and **2** are shown in Figs. 2 and 4. Compound **8** was prepared from **7** by treatment with 5% H_2_SO_4_ in methanol, as previously described^48^. Compound **8** was converted into a common precursor, IVM aglycon-1*H*-imidazole-1-carboxylate **10**, by sequentially treating **8** with (1) *tert-*butyl dimethylsilyl chloride (TBDMS-Cl) in the presence of imidazole as an activator of TBDMS-Cl and 4*-*dimethylamino pyridine (DMAP) as a nucleophilic base^48, 52^, and (2) an excess of carbonyldiimidazole (CDI, 2.0 equiv) in dry benzene/dry toluene (Fig. 2)^53^. Intermediate **10** was reacted with **6** in the presence of 1,8-diazabicyclo[5.4.0]undec-7-ene (DBU), a non-nucleophilic base, to obtain **11**. Decomposition was noticed during the purification of **11** by column chromatography. Thus, **11** was rapidly passed through the column and the residue deprotected using *p-*toluene sulphonic acid (*p-*TSA) in methanol.

**Figure 3:**
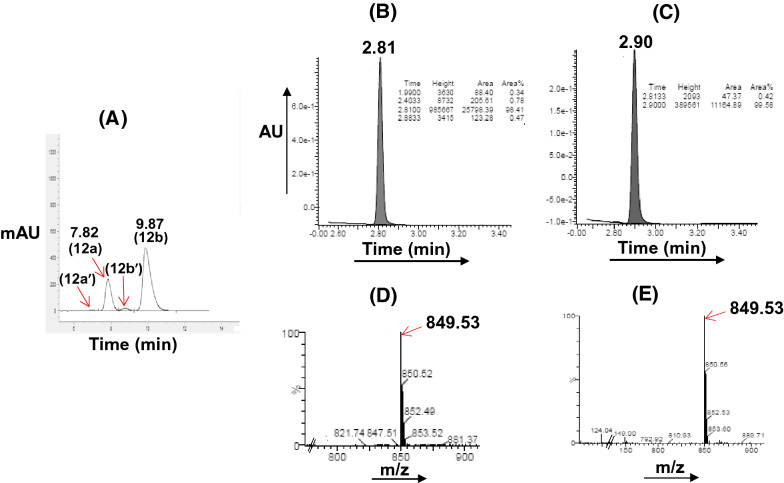
HPLC (**A**) chromatogram of a mixture of isomers of IVM-CQ **12**. UPLC chromatograms of isomers (**B**: **12a**; **C**: **12b**) after separation. UPLC-MS chromatograms: (**D**) **12a** and (**E**) **12b**.

The residue was purified by column chromatography to obtain IVM-CQ hybrid **12**, comprising of a mixture of the two inherent (R^2^ = Me and H) components in the ratio 98.87:0.6, as revealed from the high-performance liquid chromatography (HPLC) analysis (see Supplementary information, Fig. S16). Additionally, the HPLC chromatograms also indicated the presence of two isomeric peaks corresponding to each of the two inherent components. Thus, the isomeric mixture of **12**, as isolated above was subjected to preparative HPLC using reverse phase (X Select CSH C18) chromatography, and the two isomers (**12a** and **12b**/ 25.0% and 71.0%, Fig. 3) were isolated. The ultra-performance liquid chromatography (UPLC, see Supplementary information, Figs S17, S18) analysis of the two isomers revealed an analytical purity of 98.41% and 99.58% for **12a** and **12b**, respectively. The minor components of the isomeric mixture of **12** (R^2^ = H: **12a′** and **12b′**, Fig. 3) however were eliminated during preparative HPLC purification. Interestingly, both **12a** and **12b** were stable and depicted parent ion peaks at identical mass (*m/z* 849.53) suggesting **12a** and **12b** to be structural isomers, whose structures could very convincingly be assigned using high-field NMR analysis (*vide infra*).

**Figure 4:**
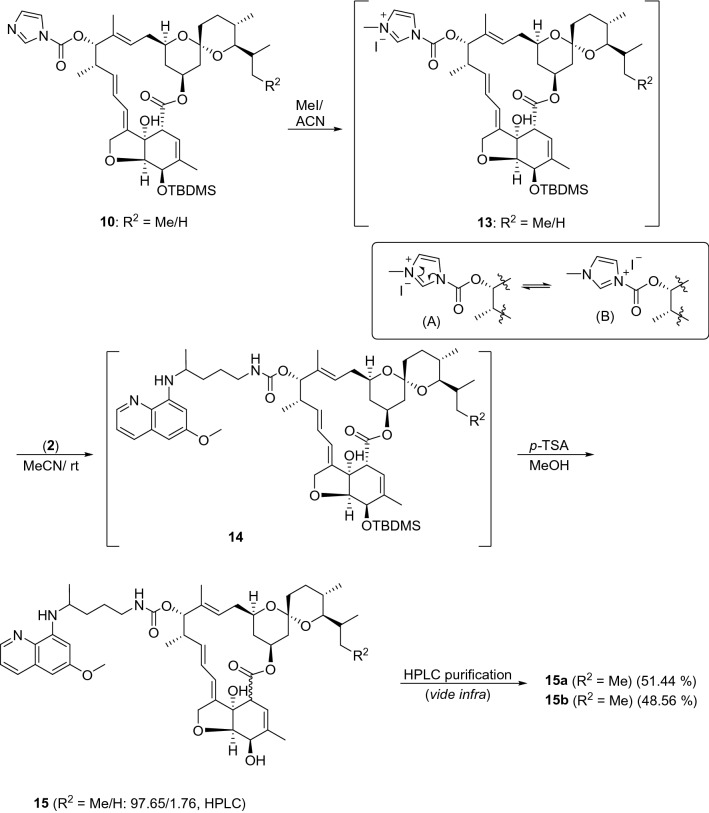
Synthesis of IVM-PQ conjugates **15** from intermediate **10** derived from **8**.

**Figure 5:**
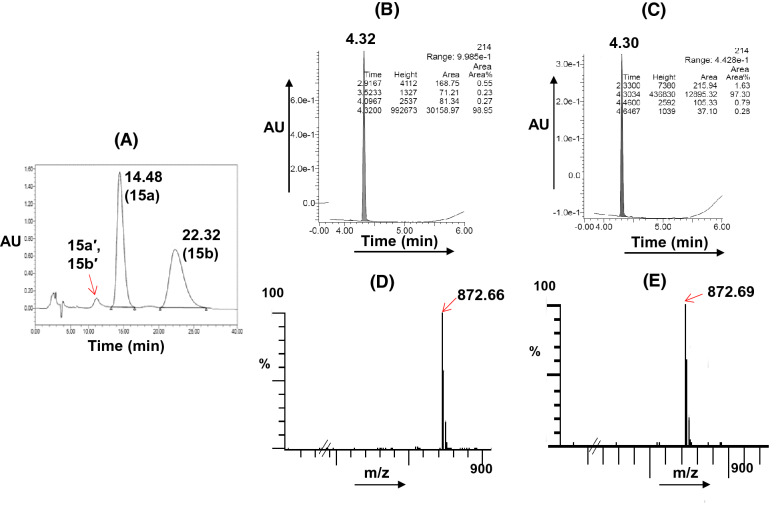
HPLC (**A**) chromatogram of original mixture of isomers of IVM-PQ **15**. UPLC chromatograms of individual isomers (**B**: **15a**; **C**: **15b**). UPLC-MS chromatograms: (**D**): **15a** and (**E**): **15b**.

For the synthesis of IVM-PQ conjugates **15**, our attempts at the use of the chemistry described in Fig. 2, by replacing **6** with **2** were not successful. Thus, treating **10** with **2** using different reaction conditions yielded the desired **14** in very poor yield. We envisaged that quaternization of the imidazole in **10** to create **13** might incorporate a better leaving group due to structural tautomerism (A ↔ B, Fig. 4) and increased yield of the desired product^54^. Thus, the reaction of intermediate **10** with iodomethane in anhydrous acetonitrile furnished the iodide salt, which was hygroscopic and washed with anhydrous ether. The reaction of **13** with **2** (*vide experimental*) straightway furnished **14** in good yield. Intermediate **14** was then deprotected using *p-*TSA to isolate the product, which was purified by column chromatography. HPLC analysis of the purified product using Chiralpak-IC column indicated the presence of the isomeric components (identified as **15a** and **15b**) of the major (R^2^ = Me) component in the ratio 51.44:48.56 (Fig. 5 and see Supplementary information, Fig. S19). The minor inherent component of **15** (R^2^ = H) was eliminated during column chromatographic purification, as it was not detected in the HPLC chromatogram. Preparative HPLC using Chiralpak-IC column was used to resolve the mixture of **15a** and **15b**, and the components (**15a**: 99.89% and **15b**: 99.74%, see Supplementary information, Figs S20, S21) were isolated in analytically pure form. However, LCMS analysis of **15a** and **15b** indicated baseline achiral impurities. Therefore, **15a** and **15b** were subjected to preparative HPLC using reverse phase (X Select CSH C18) chromatography. The UPLC (see Supplementary information, Figs S22, S23) chromatograms of the isolated **15a** and **15b** indicated 98.95% and 97.30% analytical purity, respectively.

### Characterization of isomers of 12 and 15

All compounds were unambiguously characterized by spectroscopic techniques and the spectral data is presented in the Supplementary information (Figs S1–S15, S24–S27). ^1^H NMR spectral assignments of all intermediates and compounds were performed based on their coupling connectivity as seen in the 2D ^1^H–^1^H homonuclear correlated spectroscopy (COSY) spectrum and confirmed by High Resolution Mass Spectrometry (HRMS) and/or microanalytical data. Nuclear Overhauser effect spectroscopy (NOESY) was additionally used to identify relative orientations of H in **15a** and **15b**.

The antiplasmodial activity of hybrids of IVM is strongly structure-dependent. Therefore, the unnerving yet obligatory challenge was to assign the structures of the two isomers of both **12** (**12a** and **12b**) and **15** (**15a** and **15b**). In this context, high-field NMR data presented itself as a dependable tool, as contrasting differences in the chemical shift (*δ* ppm), as well as multiplicity of the comparable protons was observed, leading to the unambiguous assignment of structures.

**Figure 6:**
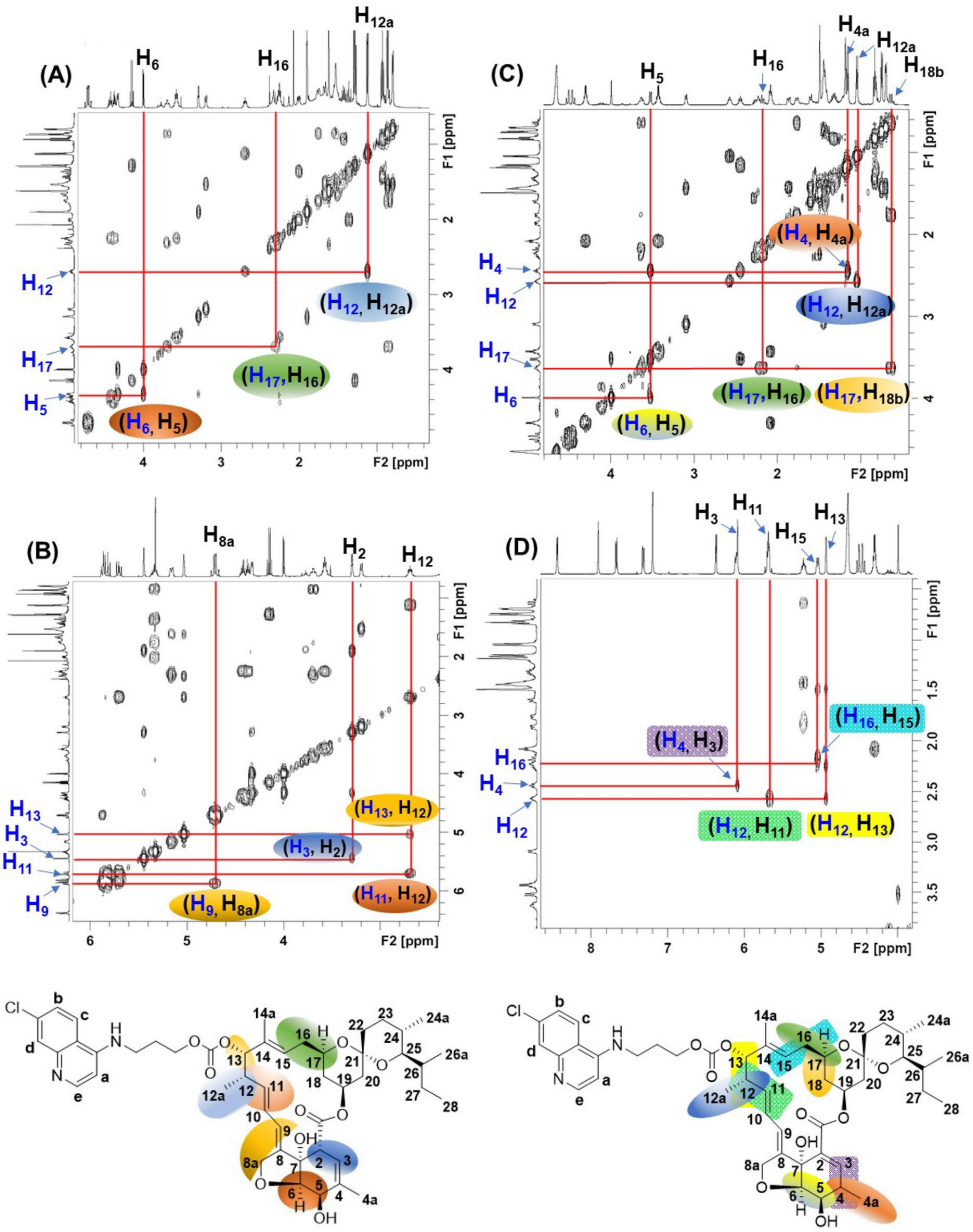
^1^H-^1^H COSY spectrum: (**A**, **B**): **12a** and (**C**, **D**): **12b**.

The ^1^H-^1^H COSY spectrum was quite useful in finding correlations leading to further simplification of the complex NMR data. In the ^1^H NMR spectrum of **12a**, the signal corresponding to C-2 H (Fig. 6) of the macrolide at *δ* 3.27 (br, 1H) was absent in the ^1^H NMR spectrum of **12b**. Instead, a 1H quintet at *δ* 2.54 (1H, *J* = 7.5 Hz) was observed in the NMR spectrum of **12b**. Based on the coupling relationship revealed by the ^1^H-^1^H COSY spectrum of **12b** (Fig. 6C), the quintet signal was assigned to the C-4 H of the macrolide. Quite convincingly, the corresponding change in the chemical shift and multiplicity of protons corresponding to C-4a of the macrolide was also observed. In ^1^H NMR of **12a**, the C-4a protons appeared as a 3H singlet at *δ* 1.87, while a 3H doublet at *δ* 1.23 (d, *J* = 7.0 Hz) was observed for the C-4a protons of **12b**. This change in the NMR spectrum hinted at the shifting of the double bond from Δ^3^ to Δ^4^ positions in the oxahydrindene ring of the macrolide. Thus, as expected, an upfield shift of the C5-H from *δ* 4.33 (d, *J* = 6.0 Hz) in **12a** to *δ* 3.61 (dd, J = 2.0, 7.5, 1H) was observed in the ^1^H NMR spectrum of **12b**. Other significant changes in the NMR spectra included: a downfield shift (Δ*δ* = 0.73 ppm) of the C3-H, and the olefin C-9 H (Δ*δ* = 0.41 ppm) of **12a** and **12b**. Based on similar proton couplings and correct mass spectral data (*vide experimental*), the structures of the isomers **12a** and **12b** were ascertained. The formation of the regioisomer **12b** could be traced back to the synthetic step where DBU was used as a base. The C-2 H in **12a** being acidic (due to the ester function) would yield a thermodynamically stable carbanion (compared to kinetically formed oxygen anions). Thus, re-protonation would yield both **12a** and **12b**.

Quite surprisingly, no significant differences were observed between the NMR spectra of isomers **15a** and **15b** (see Supplementary information, Figs S4, S5). However, the assignment of the complex signals could be readily achieved from the COSY spectra of **15a** and **15b**. For example, the cross-peaks of H_5_ with H_6_, H_12_ with H_12a_ and H_13_, H_2_ and H_3_ helped identify the coupling partners (see Supplementary information, Figs S24, S25). Given the close similarity in the NMR spectra of both **15a** and **15b**, the formation of regioisomers in analogy with **12a** and **12b** was ruled out.

**Figure 7:**
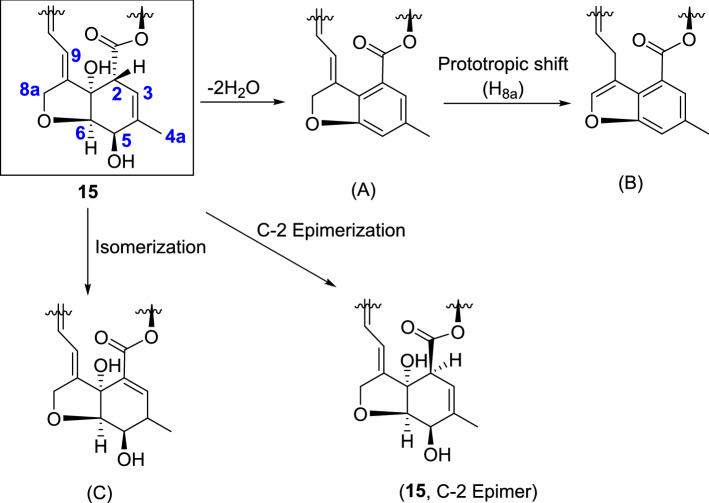
Possible chemical transformations of the oxahydrindene unit of **15**.

The oxahydrindene part of the macrolide ring of the IVM hybrids having two hydroxyl groups adjacent to sp^3^ hybridized carbons is prone to transformation into the benzenoid structure (A, Fig. 7) through double β-elimination of water, which, upon prototropic shift of a 8a-H, would result in an aromatic benzofuran ring (B). Interestingly, the spiroketal moiety of the macrolide remained intact.

**Figure 8:**
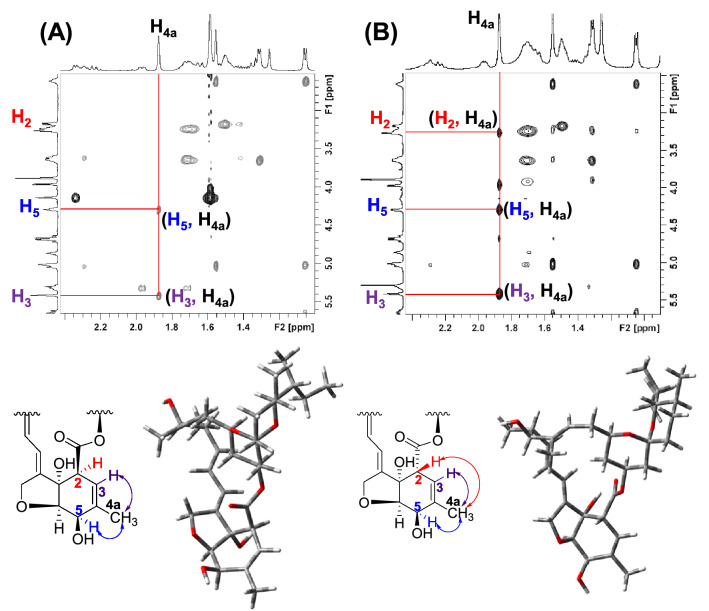
NOESY spectra (in part) of (**A**): **15a**; (**B**): **15b** showing oxahydrindene unit and the corresponding DFT optimized structures depicting aglycon units.

The presence of signals corresponding to C_2_-H, C_3_-H, C_5_-H, and C_6_-H, in the NMR spectra of both the isomers of **15** (**15a** and **15b**) led us to rule out the formation of **A-C** (Fig. 7). It was further corroborated by HRMS where a peak (*m/z* 871) corresponding to the molecular formula C_50_H_69_N_3_O_10_ of the **15a** and **15b** was observed. However, in the NOESY spectra of **15a** and **15b**, the absence of through space coupling between C_4a_-H with C_2_-H (Fig. 8 and see Supplementary information, Figs. S26, S27) in the former strongly suggested the epimerization^55, 56^ at the C-2 of the macrolide. The nOe’s between C_4a_-H and the other relevant protons, C_3_-H, C_5_-H, could be identified in both isomers.

### Antiplasmodial activity

#### In vitro activity of “second-generation” IVM hybrids against Plasmodium hepatic infection

**Figure 9:**
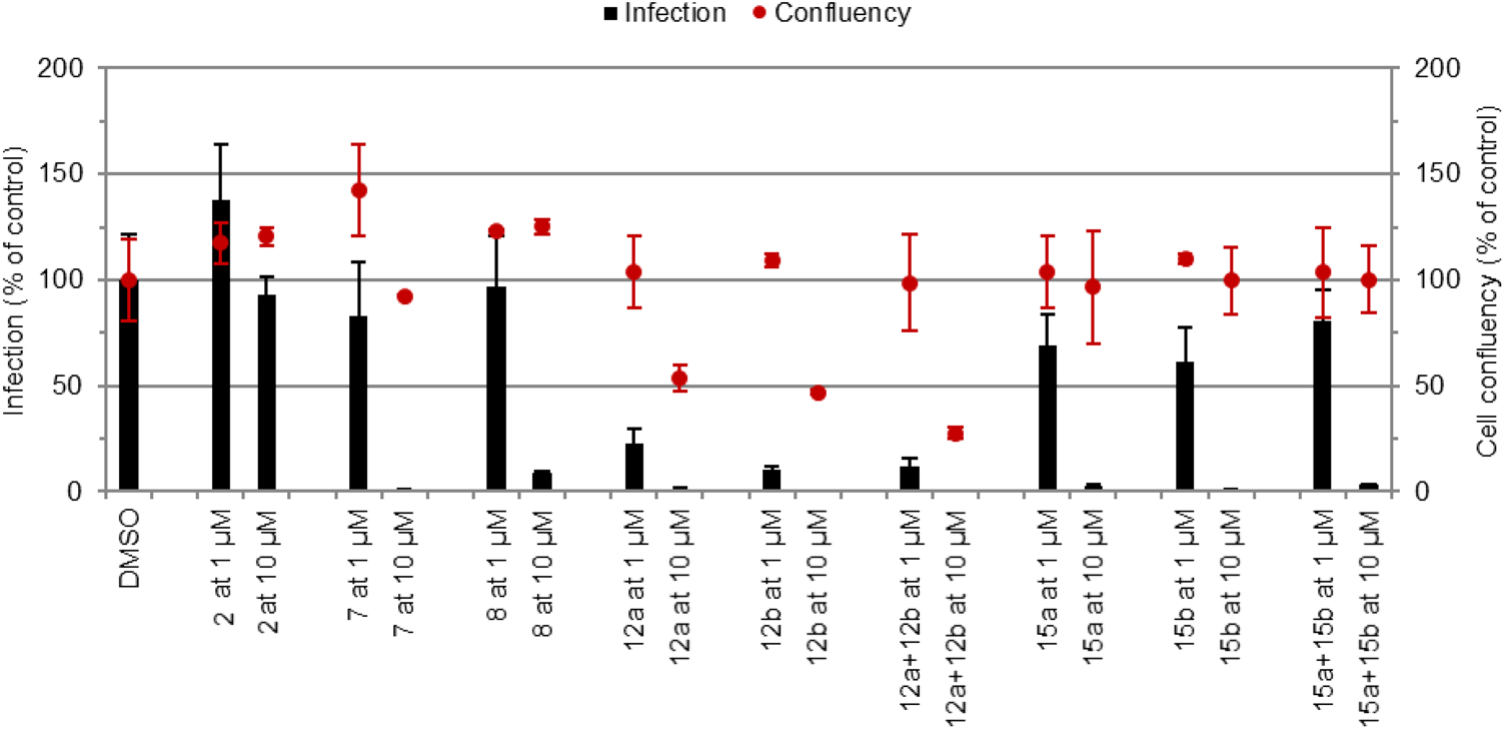
Assessment of in vitro activity of compounds against the hepatic stage of *P. berghei* infection. Total parasite load (infection scale, bars) and cell viability (cell confluency scale, dots) are shown. Results were normalized to the negative control, the drug vehicle dimethyl sulphoxide (DMSO), and are represented as mean ± SD of technical triplicates, n = 1.

IVM Hybrids **12a**,**b** and **15a**,**b** were initially screened at 10 and 1 µM for their in vitro activity against the hepatic stage of *P. berghei* infection (Fig. 9). Pristine **7**, **8**, and **2** were employed as controls in these experiments. All compounds of interest dramatically impacted infection at 10 µM. However, in the case of the CQ hybrids **12a**,**b** this was accompanied by a reduction in cell confluence, indicative of toxicity towards the host cells at this concentration.

However, hybrids **12a**,**b** were also the most active compounds at 1 µM. Given their potentially interesting activity, which was comparable to, or even higher than that of **7**, all compounds were selected for IC_50_ determination.

**Figure 10:**
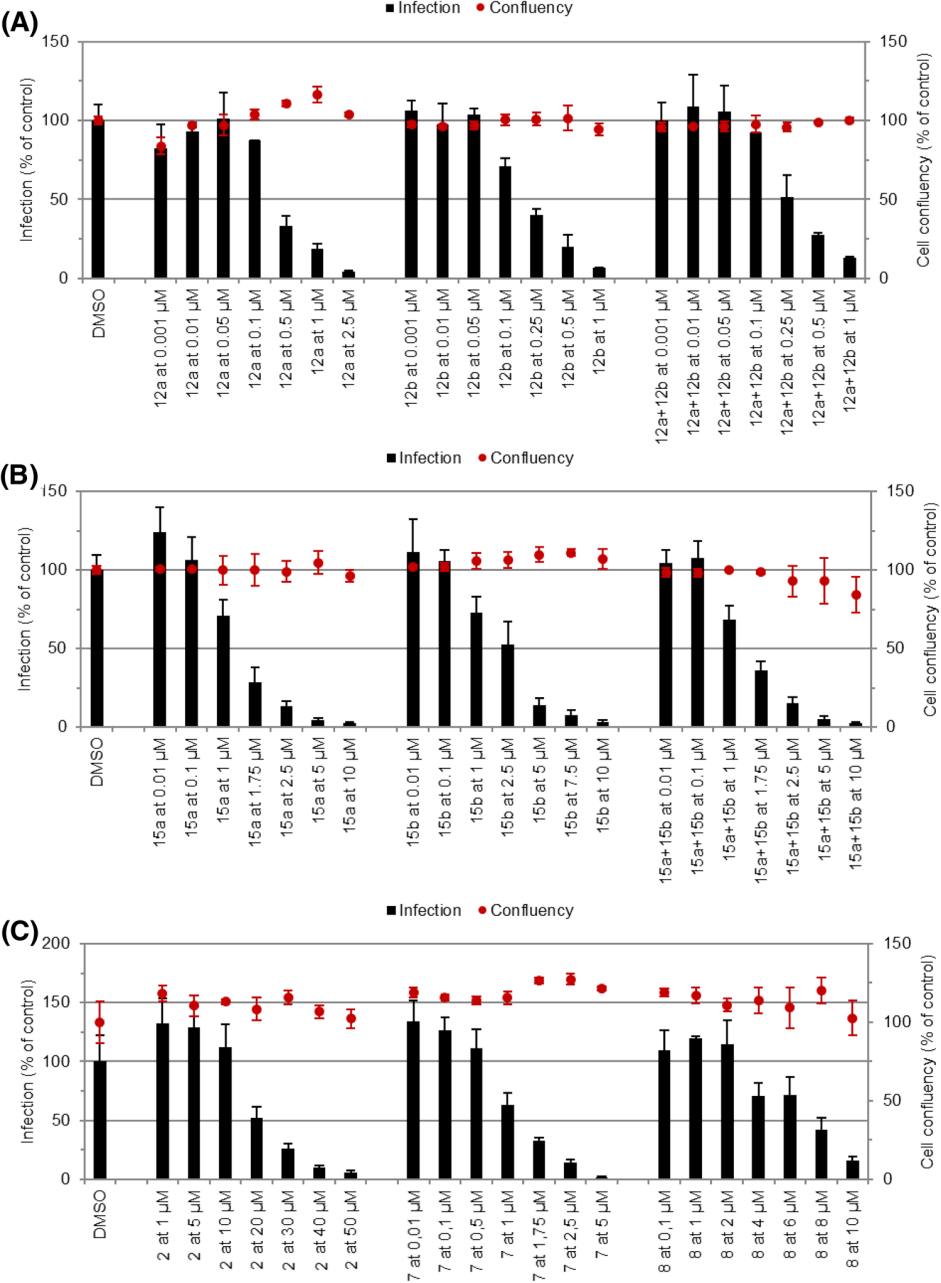
Dose-dependent response of compounds against the hepatic stage of *P. berghei* infection. The dose-dependent response of (**A**) IVM-CQ hybrids, **12a**,**b** (**B**) IVM-PQ hybrids, **15a**,**b**, and (**C**) **7**, **8** and standard drug **2** were evaluated against *P. berghei-*infected Huh7 cells. Total parasite load (infection scale, bars) and cell viability (cell confluence scale, dots) are shown, n ≥ 2. Calculated IC_50_ values are shown in Table 1.

Dose-dependent responses of each compound against *P. berghei* hepatic infection were obtained (Fig. 10), which enabled the determination of their IC_50_ (Table 1). In agreement with the data from the initial screen, CQ hybrids **12a**,**b** displayed the highest activity, with IC_50_ values ranging from 0.186 to 0.317 µM, whereas PQ hybrids **15a**,**b** were less active, with IC_50_ values ranging from 1.291 to 2.057 µM. Comparing the activity of the most potent member of the *first-generation* IVM hybrids^48^ with compound **12b**, the most active member of the current *second-generation* series, shows that the latter is nearly threefold more active than the former. The fact that the IVM hybrids are significantly more potent antiplasmodial agents than IVM warrants the chemical modification of IVM to produce antiplasmodial agents with enhanced potency. Further, the complete loss of the antiplasmodial activity of the **8** strongly suggests some role of the substitution at the C-13 position of the macrolide structure.

**Table 1:** IC_50_ values of selected compounds against the hepatic stage of *P. berghei* and the erythrocytic stage of *P. falciparum* NF54 infections.

Compound	IC_50_ (µM)^a^ *P. berghei*	IC_50_ (nM)^a^ *Pf*NF54
**12a**	0.274 ± 0.103	48.2 ± 3.1
**12b**	0.186 ± 0.025	64.8 ± 27.5
**12a + 12b**^b^	0.317 ± 0.035	74.3 ± 33.0
**15a**	1.291 ± 0.042	ND
**15b**	2.057 ± 0.159	ND
**15a + 15b**^b^	1.360 ± 0.006	ND
**7**	1.321 ± 0.011	359.6 ± 65.7
**8**	6.375 ± 0.909	ND
**2**	8.428 ± 3.389^57^	ND
**1**	ND	23.7 ± 10.1^48^

^a^Results are represented as mean ± SD, n ≥ 2. ND: not determined.

^b^Original isolated mixture, before HPLC.

#### In vitro activity against P. falciparum erythrocytic infection

**Figure 11:**
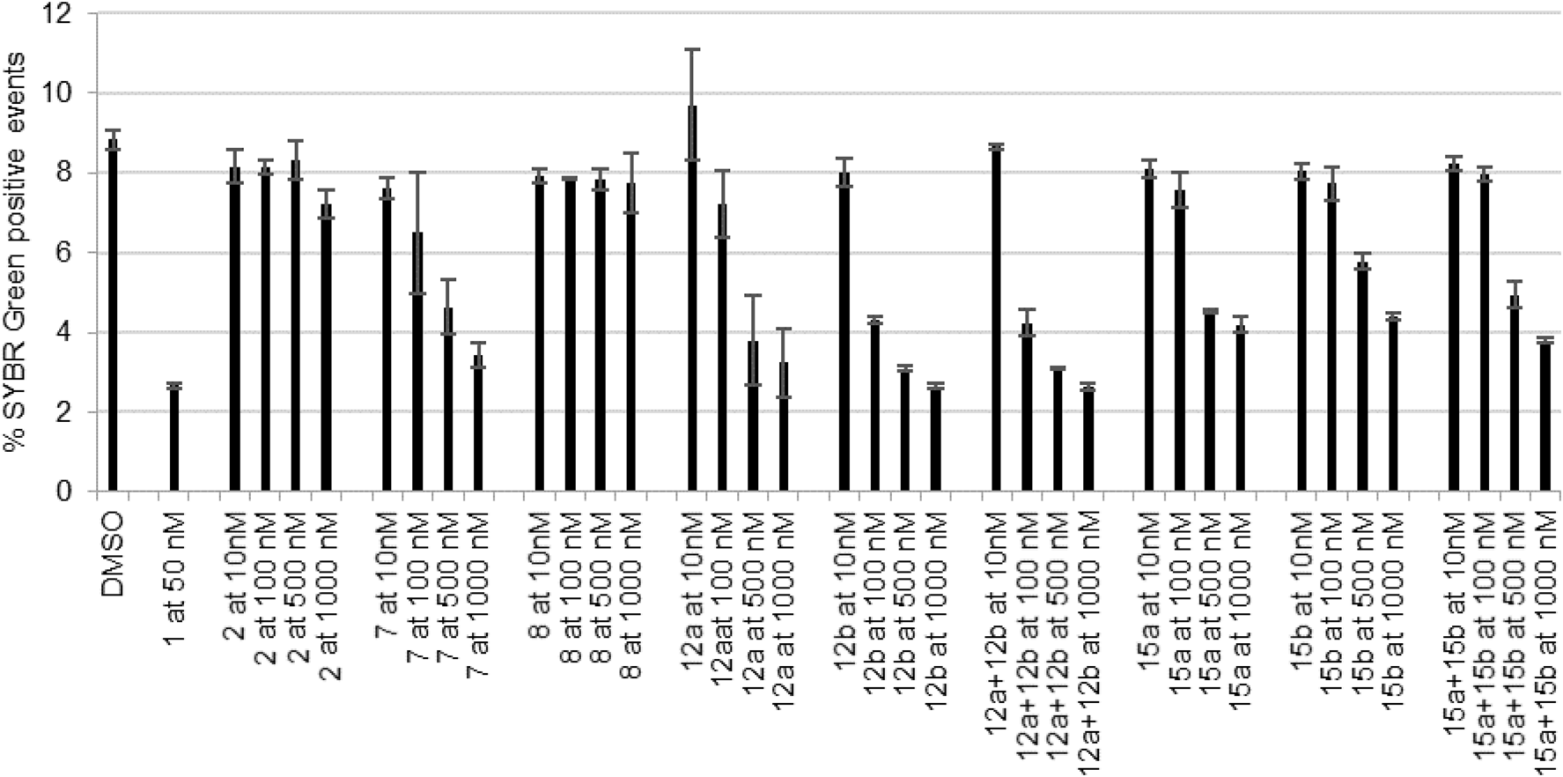
Assessment of in vitro activity of compounds against the blood stage of *P. falciparum* infection. Percentage of SYBR Green positive events (parasitemia) against each compound at 1000 nM, 500 nM, 100 nM, and 10 nM is shown. Chloroquine (CQ) at 50 nM was used as the positive control. Results were compared with the negative control (DMSO) and are represented as the mean ± SD of technical triplicates, n = 1.

To assess their activity against the blood stage of *P. falciparum* (*Pf*NF54) infection, compounds were initially screened at 1000, 500, 100, and 10 nM (Fig. 11). Compounds **7, 8**, **2**, and **1** were employed as controls. Compound **8** and **2** were not active against the parasite as shown by comparison with the DMSO control. Similar to what was observed for the hepatic stage, CQ hybrids **12a,b** showed the highest activity against *P. falciparum* blood stages. For that reason, **12a**, **12b**, mixture **12a** + **12b**, and **7** were selected for IC_50_ determination.

**Figure 12:**
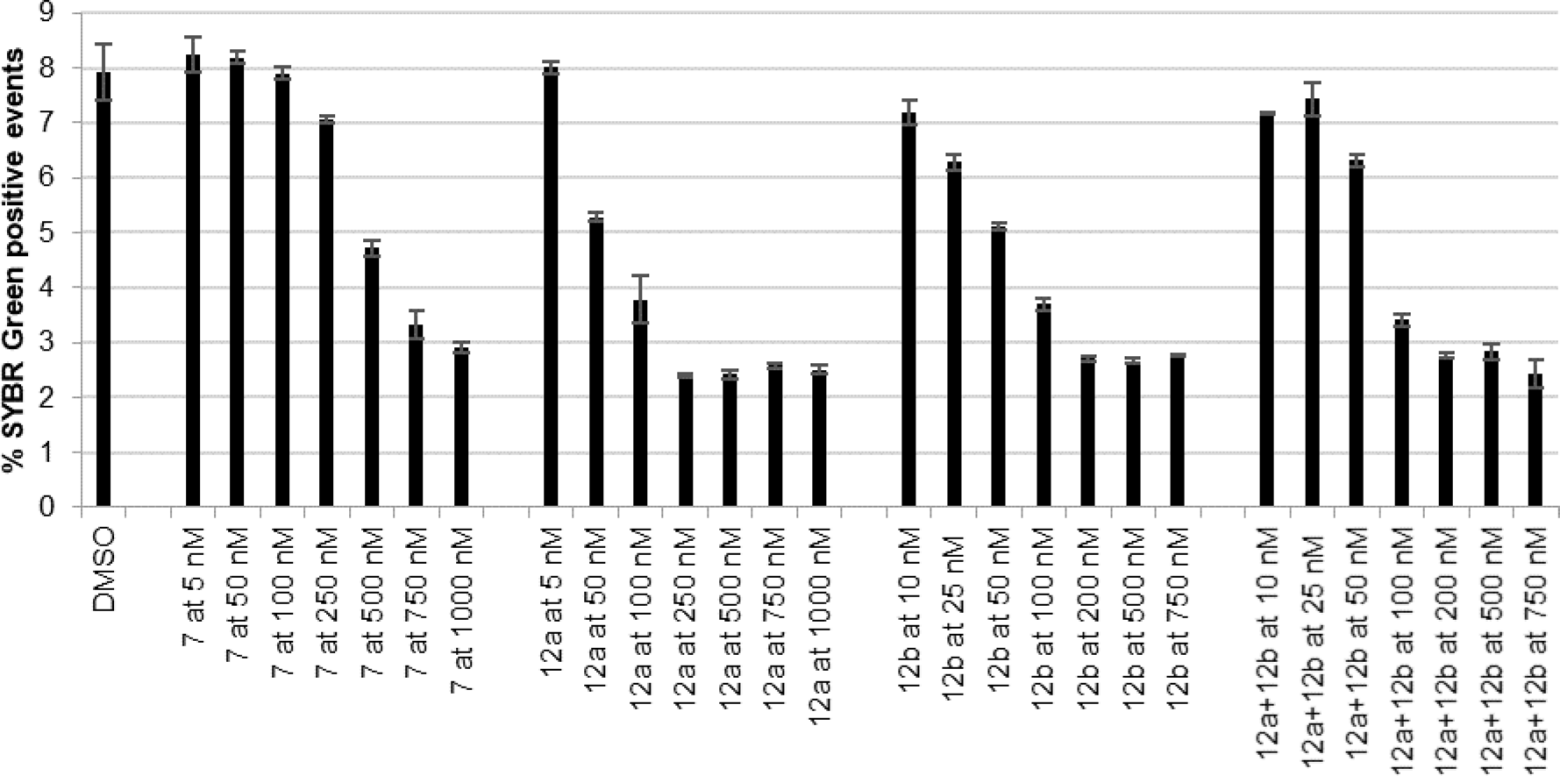
Inhibition of *P. falciparum* in the presence of different concentrations of **12a** + **12b** mixture, **12a**, **12b,** and **7**. A ring stage synchronized culture of *P.* *falciparum* (*Pf*NF54) was incubated for 48 h with increasing concentrations of the compounds, DMSO (vehicle control). The percentage of SYBR Green positive events per concentration is represented. DMSO is represented as a negative control. Each time point represents the mean value of triplicate measurements (± one SD), n = 3.

Dose-dependent values of the % of SYBR Green-positive events were obtained at selected concentrations (Fig. 12) for IC_50_ calculation (Table 1). CQ hybrids, **12a**, **12b** displayed IC_50_ values between 48.2 and 74.3 nM, an activity lower than that previously determined for the CQ control (23.7 nM ± 10.1)^48^. Compound **7** was the least active compound tested, with an estimated IC_50_ of 359.6 nM.

It is interesting to note that the IVM-PQ hybrids **15a** and **15b** display lower activity than their CQ counterparts **12a** and **12b** against both stages of *Plasmodium* infection. This is unsurprising in what concerns the parasite’s blood stages, since **1** is a known blood stage antiplasmodial, whereas **2** does not display significant activity against this stage of the parasite’s life cycle.

The fact that **12a** and **12b** are also more active than **15a** and **15b** against hepatic infection is somewhat puzzling. However, it should be noted that the in vitro hepatic stage antiplasmodial activity of **7** (~ 2 μM) is higher than that of **2** (~ 10 μM) and, as such, the IVM moiety of the PQ hybrids **15a** and **15b** is the main contributor towards hepatic stage activity. The data (Table 1) suggests that the hepatic stage activity is potentiated by CQ analogue more than PQ in the hybrids of IVM. Evidently, when hybridized with IVM, the enhanced synergization of the former leads to the enhanced hepatic stage antiplasmodial activity, whereas such enhancement is absent in the PQ hybrids **15a** and **15b**, where the activity primarily results from the IVM moiety.

## Conclusions

The *second-generation* IVM hybrids synthesized through molecular hybridization of **7** with the CQ analogue **6** and antiplasmodial drug **2** display higher potency against the hepatic and blood stages of *Plasmodium* infection than their *first-generation* counterparts. IVM-CQ hybrids **12a**,**b** were the most active compounds against *P. berghei* hepatic infection in vitro. Interestingly, IVM hybrids [IC_50_ = 0.274 µM (**12a**) and 0.186 µM (**12b**)] displayed higher activity against *P. berghei* infection than **7** (IC_50_ = 1.321 µM) and the standard liver-stage drug, **2** (IC_50_ = 8.428 µM). Surprisingly, the IVM-PQ hybrids were less active [IC_50_ = 1.291 µM (**15a**) and 2.057 µM (**15b**)] against *P. berghei* hepatic infection than the IVM-CQ hybrids.

The blood-stage antiplasmodial activity of the compounds against the *P. falciparum* NF54 strain was significantly higher than that observed against the hepatic infection. Compound **12a** was the most active (IC_50_ = 48.2 nM) displaying over sevenfold higher potency than the pristine IVM, and more active than the comparable member of the series of the *first-generation* IVM hybrids, emphasizing the advantage of the hybridization approach described here. IVM-PQ hybrids were not active enough to determine IC_50_ values.

Compound **8** displayed the lowest activity of all the compounds synthesized and paralleled the trend observed in the series of the *first-generation* IVM hybrids. Additionally, we evaluated the activity of the mixture of isomers in order to determine whether it is necessary to isolate the isomers. Reassuringly, our results showed that the in vitro antiplasmodial activities against both the hepatic and blood-stage infections by *Plasmodium* were generally lower than those of the individual isomers.

Overall, our data highlights the enhancement of the antiplasmodial activity of these structurally modified compounds through appending hybrid partners at C-13 of the macrolide unit. Work towards designing and synthesizing additional structurally modified IVM hybrids is currently in progress.

## Experimental

### General

All liquid reagents were dried/purified following recommended drying agents and/or distilled over 4 Å molecular sieves. CH_3_CN was dried by refluxing over P_2_O_5_. DCM was dried over fused calcium chloride. TBDMS-Cl, DMAP, and CDI were bought from Spectrochem. Imidazole was bought from Sigma Aldrich. K_2_CO_3_ was dried overnight in the furnace. ^1^H NMR and ^13^C NMR spectra were recorded on Bruker Biospin Avance III HD at 500 MHz and JEOL-FT NMR-AL at 400 MHz with TMS as an internal standard using CDCl_3_ as deuterated solvent. Data are reported as follows: chemical shift in *δ* (ppm), integration, multiplicity (s = singlet, d = doublet, t = triplet, m = multiplet), coupling constant *J* (Hz). High-Resolution Mass spectra were recorded on a Bruker LC–MS MICROTOF II spectrometer. IR spectra were recorded on Agilent Technologies Cary 630 FTIR spectrometer. HPLC was performed in Reverse Phase mode using Agilent 1260 Infinity series HPLC (Agilent Technologies, USA) equipped with Quaternary Pump VL (G1311C) and degasser, 1260 ALS auto sampler (G1329B), and 1260 DAD VL detector (G1315D). The column used was the ZORBAX Eclipse C18 column (4 × 100 mm) (Agilent Technologies, USA). The mobile phase was comprised of a mixture of acetonitrile-methanol–water (49.2:32.8:18 *v/v/v*) and the flow rate was set to 1.0 mL/min. Agilent 1260 Infinity auto purification system with 1260 DAD VL-UV detector was used for preparative HPLC analysis. For reverse-phase prep purification of **12**, the mobile phase was comprised of a mixture of 5 mM ammonium acetate in water-acetonitrile (10:90 *v/v*) and the flow rate was set to 18.0 mL/min. The column used was X SELECT CSH C18 (10 × 250 mm, 10 μm). For chiral purification of **15**, the mobile phase comprised of a mixture of ethanol-0.1% TEA in *n*-hexane (30:70 *v/v*). The column used was Chiralpak-IC (21 × 250 mm, 5 µm) and the flow rate was set to 1.0 mL/min. For reverse-phase prep purification, the mobile phase was comprised of a mixture of 5 mM ammonium acetate in water-acetonitrile (10:90 *v/v*) and the flow rate was set to 18.0 mL/min. The column used was X SELECT CSH C18 (10 × 250 mm, 10 μm). DFT optimization of geometry was done using Gaussian09 software (STO 3G basis set). GaussView 5.0.9 molecular software was used for visualizing the optimized structures. All tested compounds are > 95% pure by HPLC/UPLC analysis. Optical rotations were determined with an AUTOPOL IV polarimeter at 25 °C using sodium D light in methanol (HPLC grade). Concentration “*c*” is depicted in g/mL.

### Synthesis of 10

To the suspension of CDI (0.23 g, 1.4 mmol) in dry toluene (10 ml), a solution of **9** (0.5 g, 0.7 mmol) in dry toluene (5 ml) was added dropwise. The resulting mixture was stirred at room temperature for 12 h. The reaction mixture was poured into water and extracted with chloroform (2 × 20 ml). The organic extract was washed with water, dried over anhydrous sodium sulfate, filtered, and evaporated to obtain a brown oil. The crude product was purified by column chromatography over silica gel (60–120 mesh) using hexane/ethyl acetate (75:25, *v/v*) as eluent to afford **10** as a white solid in 73% yield. IR. 2929, 1766, 1461, 1394, 1282, 1177 cm^−1^. ^1^H NMR (500 MHz, CDCl_3_, 25 °C): *δ* 8.22 (s, 1H, imidazole), 7.49 (br, 1H, imidazole), 7.14 (br, 1H, imidazole), 5.81–5.87 (m, 2H, H_9_, H_10_), 5.61–5.69 (1H, m, H_11_), 5.27–5.33 (m, 3H, H_19_, H_3_, H_13_), 5.07 (t, 1H, *J* = 7.5 Hz, H_15_), 4.72 (ABq, 2H,* J* = 15.0 Hz, 2 × H_8a_), 4.44 (br, 1H, H_5_), 4.19 (s, 1H, C_7_-OH), 3.84 (d, 1H, *J* = 5.5 Hz, H_6_), 3.57–3.63 (m, 1H, H_17_), 3.38 (br, 1H, H_2_), 3.14 (d, 1H, *J* = 8.0 Hz, H_25_), 2.74–2.81 (m, 1H, H_12_), 2.28 (t, 2H, *J* = 8.5 Hz, 2 × H_16_), 2.02 (dd, 1H, *J* = 4.5, 8.0 Hz, H_18a_), 1.79 (s, 3H, 3 × H_4a_), 1.31–1.71 (m, 13H, 2 × H_22_, 2 × H_23_, H_24_, H_26_, 2 × H_27_, 2 × H_20_, 3 × H_14a_), 1.15 (d, 3H, *J* = 7.0 Hz, 3 × H_12a_), 0.93 (s, 9H, (CH_3_)_3_C), 0.80–0.86 (m, 7H, 3 × H_28_, 3 × H_26a_, H_18b_), 0.76 (d, 3H, *J* = 5.0 Hz, 3 × H_24a_), 0.14 (s, 6H, (CH_3_)_2_Si). ^13^C NMR (100 MHz, CDCl_3_, 25 °C): *δ* 173.9, 148.1, 142.3, 137.6, 137.2, 134.5, 133.7, 131.1, 126.4, 118.7, 118.7, 117.3, 116.9, 97.6, 83.3, 80.3, 80.1, 77.3, 69.5, 68.7, 67.9, 66.9, 45.7, 41.3, 39.2, 36.8, 35.8, 35.5, 34.2, 31.2, 28.1, 27.4, 25.9, 20.1, 18.9, 18.5, 17.5, 14.8, 12.5, 11.9, -4.5, -4.8. HRMS: *m/z* [M + Na]^+^ for C_44_H_66_N_2_O_9_Si, calculated 817.4430; observed 817.3509.

### Synthesis of 12a and 12b

DBU (0.086 g, 0.57 mmol) was added to the suspension of **6** (0.02 g, 0.57 mmol) in 5 ml dry DCM and the resulting clear solution was stirred at room temperature for 15 min before dropwise addition of a solution (5 ml) of **10** (0.300 g, 0.38 mmol) in DCM. The reaction mixture was stirred at room temperature for 12 h. Upon completion, the reaction mixture was poured into water and extracted with water. The organic layer was dried over anhydrous sodium sulfate and the solvent was evaporated to brown oil (**11**, 0.310 g). For deprotection, 10 ml solution of *p*-TSA in methanol (0.02 g mL^-1^) was added to the solution of **11** (0.310 g) in methanol (10 ml) dropwise. The reaction mixture was stirred for 30 min at room temperature. Upon completion, DCM (30 ml) was added to the reaction mixture and washed with aqueous sodium bicarbonate, water, dried over anhydrous sodium sulfate, and the solvent was evaporated to give the brown crude product which was purified by column chromatography over silica gel (60–120 mesh) using chloroform/methanol (95:5, *v/v*) as the eluent afford **12** as a white solid in 39.9% yield. Characteristic data of isolates of **12** obtained after prep purification is given below.

**12a** White solid. IR. 3302, 3255, 2914, 1729, 1587, 1468, 1177 cm^-1^. ^1^H NMR (500 MHz, CDCl_3_, 25 °C): *δ* 8.51 (d, 1H, *J* = 5.0 Hz, ArH), 7.97 (br, 1H, ArH), 7.73 (d, 1H, *J* = 9.0 Hz, ArH), 7.38 (dd, 1H, *J* = 2.0, 7.0 Hz, ArH), 6.43 (d, 1H, *J* = 5.5 Hz, ArH), 5.67–5.87 (m, 3H, H_9_, H_10_, H_11_), 5.65 (br, 1H, NH), 5.42 (s, 1H, H_3_), 5.28–5.35 (m, 1H, H_19_), 5.16 (d, 1H, *J* = 3.5, 7.0 Hz, H_15_), 5.02 (s, 1H, H_13_), 4.72 (ABq, 2H, *J* = 2.0, 8.0, 12.5 Hz, 2 × H_8a_), 4.53 (br, 1H, C_7_-OH), 4.33–4.41 (m, 2H, CH_2_O-), 4.30 (d, 1H,* J* = 6.0 Hz, H_5_), 3.98 (d,* J* = 6.0 Hz, H_6_), 3.64–3.69 (m, 1H, H_17_), 3.45–3.52 (m, 2H, CH_2_NH), 3.27 (br, 1H, H_2_), 3.17 (d, *J* = 8.0, H_25_), 2.64–2.71 (m, 1H, H_12_), 2.24–2.33 (m, 2H, 2 × H_16_), 2.11–2.17 (m, 2H, CH_2_), 2.0 (dd, 1H, *J* = 4.5, 8.0 Hz, H_18a_), 1.87 (s, 3H, 3 × H_4a_), 1.72–1.75 (m, 2H, H_20a_, C_5_-OH), 1.66 (d, 1H, *J* = 12.5 Hz, H_20b_), 1.60 (s, 3H, 3 × H_14a_), 1.25–1.52 (m, 8H, 2 × H_22_, 2 × H_23_, H_24_, H_26_, 2 × H_27_), 1.12 (d, 3H,* J* = 6.5 Hz, 3 × H_12a_), 0.91 (t, 3H, *J* = 7.5 Hz, 3 × H_28_), 0.84 (d, 3H, *J* = 7.0 Hz, 3 × H_26a_), 0.80–0.83 (m, 1H, H_18b_), 0.77 (d, 3H,* J* = 5.5 Hz, 3 × H_24a_). ^13^C NMR (125 MHz, CDCl_3_, 25 °C): *δ* 173.6, 155.1, 153.4, 141.0, 140.9, 140.7, 137.9, 135.9, 134.2, 125.7, 125.7, 121.1, 120.0, 118.1, 118.0, 117.0, 98.8, 97.5, 83.1, 80.3, 79.1, 77.1, 68.5, 68.4, 67.7, 67.0, 66.5, 45.7, 41.2, 39.7, 39.2, 36.8, 35.8, 35.5, 34.2, 31.2, 28.0, 27.9, 27.4, 19.9, 18.7, 17.4, 14.6, 12.5, 11.9. HRMS: *m/z* [M + H]^+^ for C_47_H_61_ClN_2_O_10_, calculated 849.4087; observed 849.4038. UPLC purity: 98.41%. t_R_ (HPLC) = 7.82 min.

**12b** White solid. IR. 3302, 3235, 2914, 1729, 1468, 1177 cm^-1^. ^1^H NMR (500 MHz, CDCl_3_, 25 °C): *δ* 8.51 (d, 1H, *J* = 5.5 Hz, ArH), 7.97 (br, 1H, ArH), 7.73 (d, 1H, *J* = 9.0 Hz, ArH), 7.40 (dd, 1H, *J* = 1.5, 7.5 Hz, ArH), 6.44 (d, 1H, *J* = 5.5 Hz, ArH), 6.15–6.19 (m, 2H, H_9_, H_3_), 5.71–5.80 (m, 2H, H_10_, H_11_), 5.65 (br, 1H, NH, D_2_O Exchangeable), 5.26–5.32 (m, 1H, H_19_), 5.12 (d, 1H, *J* = 10.5 Hz, H_15_), 5.00 (s, 1H, H_13_), 4.85 (br, 1H, C_7_-OH, D_2_O Exchangeable), 4.60 (ABq, 2H, *J* = 2.0, 12.0, 18.5 Hz, 2 × H_8a_), 4.33–4.41 (m, 2H, CH_2_O), 4.06 (br, H_6_), 3.67–3.74 (m, 1H, H_17_), 3.61 (dd, 1H, *J* = 2.0, 7.5 Hz, H_5_), 3.46–3.54 (m, 2H, CH_2_NH), 3.17 (d, *J* = 8.5 Hz, H_25_), 2.61–2.67 (m, 1H, H_12_), 2.54 (quintet, 1H, *J* = 7.5 Hz, H_4_), 2.20–2.36 (m, 2H, 2 × H_16_), 2.18 (quintet, 2H, *J* = 6.5 Hz, CH_2_), 1.95 (dd, 1H, J = 4.5, 7.5 Hz, H_18a_), 1.82–1.85 (m, 2H, H_20a_, C_5_-OH, D_2_O Exchangeable), 1.68 (d, 1H, *J* = 11.5 Hz, H_20b_), 1.56 (s, 3H, 3 × H_14a_), 1.33–1.52 (m, 8H, 2 × H_22_, 2 × H_23_, H_24_, H_26_, 2 × H_27_), 1.23 (d, 3H, J = 7.0 Hz, 3 × H_4a_) 1.12 (d, 3H, *J* = 6.5 Hz, 3 × H_12a_), 0.91 (t, 3H, *J* = 7.5 Hz, 3 × H_28_), 0.82 (d, 3H, *J* = 7.0 Hz, 3 × H_26a_), 0.77 (d, 3H, *J* = 5.5 Hz, 3 × H_24a_), 0.67–0.74 (m, 1H, H_18b_). ^13^C NMR (125 MHz, CDCl_3_, 25 °C): *δ* 168.6, 155.1, 140.3, 138.8, 136.0, 133.9, 129.8, 126.4, 125.7, 122.5, 121.1, 117.9, 117.0, 98.8, 97.4, 83.3, 82.9, 78.5, 72.3, 68.7, 68.2, 66.9, 66.4, 60.4, 53.4, 40.3, 39.7, 39.1, 36.8, 35.8, 35.5, 34.5, 33.4, 31.2, 29.7, 28.0, 27.9, 27.4, 21.0, 18.7, 17.4, 16.9, 14.6, 14.1, 12.5, 11.8. HRMS: *m/z* [M + H]^+^ for C_47_H_61_ClN_2_O_10_, calculated 849.4087; observed 849.3968. UPLC purity: 99.58%. t_R_ (HPLC) = 9.87 min.

### Synthesis of 13

Compound **10** (0.200 g**,** 0.25 mmol) was dissolved in 2 ml of dry ACN, and MeI (0.08 ml, 1.25 mmol) was added. The resulting colorless solution was stirred at 40 °C for 2 h. Upon completion, the excess of ACN was evaporated under reduced pressure to yield yellow solid **13**, which was used as such without any further purification in the subsequent reactions as it displayed significant degradation in contact of air.

### Synthesis of 15a and 15b

IVM-based intermediate **13** (0.100 g, 0.12 mmol) was dissolved in dry ACN (5 ml). To this, 5 ml solution of neutralized PQ (**2**, 0.048 g, 0.18 mmol) in dry ACN was added. The reaction mixture was stirred for 4–6 h in dark. Upon the completion, the excess of ACN was evaporated under vacuum, followed by the addition of DCM (30 ml). The resulting solution was washed with water, dried over anhydrous sodium sulfate, evaporated to obtain a dark brown oil (**14**, 0.130 g). For deprotection, 10 ml solution of *p*-TSA in methanol (0.02 g mL^-1^) was added to the solution of a **14** (0.130 g) in methanol (10 ml). The reaction mixture was stirred for 30 min at room temperature. Upon completion, DCM (30 ml) was added to the reaction mixture and washed with aqueous sodium bicarbonate, water, dried over anhydrous sodium sulfate and the solvent was evaporated to give the brown crude product which was purified by column chromatography over silica gel (60–120 mesh) using hexane/ethyl acetate (60:40, *v/v*) as the eluent afford to **15** as a white solid in 48% yield. Characteristic data of isolates of **15** obtained after prep purification is given below.

**15a** White solid. IR. 3302, 3235, 2914, 1729, 1617, 1520, 1468, 1177 cm^-1^. ^1^H NMR (500 MHz, CDCl_3_, 25 °C): *δ* 8.53 (dd, 1H, *J* = 3.0 Hz, ArH), 7.93 (d, 1H, *J* = 8.0 Hz, ArH), 7.30–7.32 (m, 1H, ArH), 6.34 (d, 1H, *J* = 2.5 Hz, ArH), 6.29 (d, 1H, *J* = 2.0 Hz, ArH), 6.00 (s, 1H, Ar–NH, D_2_O Exchangeable), 5.61–5.84 (m, 3H, H_9_, H_10_, H_11_), 5.41 (s, 1H, H_3_), 5.29–5.35 (m, 1H, H_19_), 5.01–5.04 (m, 2H, H_15_, H_13_), 4.87 (br, 1H, NH, D_2_O Exchangeable), 4.71 (ABq, 2H,* J* = 9.5, 14.5 Hz, 2 × H_8a_), 4.30 (d, 1H, *J* = 6.0 Hz, H_5_), 4.22 (br, 1H, C_7_-OH, D_2_O Exchangeable), 3.97 (d, *J* = 6.0 Hz, H_6_), 3.88 (s, 3H, OCH_3_), 3.59–3.64 (m, 2H, H_17_, CHCH_3_), 3.23–3.26 (m, 3H, H_2_, CH_2_), 3.18 (d, *J* = 8.5 Hz, H_25_), 2.59–2.62 (m, 1H, H_12_), 2.20–2.36 (m, 3H, 2 × H_16_, C_5_-OH, D_2_O Exchangeable), 1.98 (dd, 1H, *J* = 4.0, 7.5 Hz, H_18a_), 1.87 (s, 3H, 3 × H_4a_), 1.33–1.72 (m, 20H, 2 × H_22_, 2 × H_23_, H_24_, H_26_, 2 × H_27_, 2 × H_20_, 3 × H_14a_, CHCH_3_, CH_2_CH_2_), 1.06 (m, 3H,* J* = 7.0 Hz, 3 × H_12a_), 0.91 (t, 3H, *J* = 7.5 Hz, 3 × H_28_), 0.84 (d, 3H, *J* = 7.0 Hz, 3 × H_26a_), 0.80–0.82 (m, 1H, H_18b_), 0.77 (d, 3H,* J* = 5.5 Hz, 3 × H_24a_). ^13^C NMR (125 MHz, CDCl_3_, 25 °C): *δ* 173.7, 159.4, 155.9, 144.9, 144.3, 140.2, 137.9, 137.5, 135.4, 134.8, 129.9, 124.9, 121.9, 120.3, 118.1, 117.4, 97.5, 96.8, 91.7, 80.3, 79.1, 79.0, 76.9, 68.6, 68.5, 67.7, 67.2, 55.2, 47.8, 45.7, 41.3, 41.1, 39.2, 36.8, 35.8, 35.5, 34.2, 33.9, 31.2, 28.0, 27.3, 26.7, 20.6, 19.9, 18.7, 17.4, 14.6, 12.5, 12.0. HRMS: m/z [M + H]^+^ for C_50_H_69_N_3_O_10_, calculated 872.5055; observed 872.5011. UPLC purity: 98.95%. t_R_ (HPLC) = 14.48 min. [α]_D_^25^ + 26.667° (*c* 0.0015, CH_3_OH).

**15b** White solid. IR. 3302, 3235, 2914, 1729, 1595, 1468, 1177 cm^-1^. ^1^H NMR (500 MHz, CDCl_3_, 25 °C): *δ* 8.53 (d, 1H,* J* = 3.5 Hz, ArH), 7.93 (d, 1H, *J* = 8.0 Hz, ArH), 7.29–7.32 (m, 1H, ArH), 6.34 (s, 1H, ArH), 6.28 (s, 1H, *J* = 2.0 Hz, ArH), 6.00 (s, 1H, Ar–NH, D_2_O Exchangeable), 5.63–5.84 (m, 3H, H_9_, H_10_, H_11_), 5.41 (s, 1H, H_3_), 5.29–5.33 (m, 1H, H_19_), 5.01–5.04 (m, 2H, H_15_, H_13_), 4.86 (br, 1H, NH, D_2_O Exchangeable), 4.71 (ABq, 2H,* J* = 9.0, 14.5 Hz, 2 × H_8a_), 4.30 (d, 1H,* J* = 5.0 Hz, H_5_), 3.89 (s, 3H, OCH_3_), 3.97 (d, *J* = 6.0 Hz, H_6_), 3.62–3.63 (m, 2H, H_17_, CHCH_3_), 3.21–3.26 (m, 3H, H_2_, CH_2_), 3.18 (d, *J* = 9.0 Hz, H_25_), 2.59–2.61 (m, 1H, H_12_), 2.19–2.36 (m, 3H, 2 × H_16_, C_5_-OH D_2_O Exchangeable), 1.97 (m, 1H, H_18a_), 1.87 (s, 3H, 3 × H_4a_), 1.33–1.72 (m, 20 H, 2 × H_22_, 2 × H_23_, H_24_, H_26_, 2 × H_27_, 2 × H_20,_ 3 × H_14a_, CHCH_3_, CH_2_CH_2_), 1.05 (d, 3H, *J* = 6.5 Hz, 3 × H_12a_), 0.89 (t, 3H, *J* = 7.0 Hz, 3 × H_28_), 0.84 (d, 3H,* J* = 6.0 Hz, 3 × H_26a_), 0.81–0.82 (m, 1H, H_18b_), 0.77 (d, 3H, *J* = 4.5 Hz, 3 × H_24a_). ^13^C NMR (125 MHz, CDCl_3_, 25 °C): *δ* 173.7, 159.4, 155.9, 144.9, 144.4, 140.2, 137.9, 135.4, 134.8, 129.9, 124.9, 121.9, 120.3, 118.1, 117.4, 97.5, 96.7, 91.7, 80.3, 79.1, 79.0, 75.0, 68.6, 68.5, 67.7, 67.2, 55.2, 47.8, 45.7, 41.2, 41.1, 39.2, 36.8, 35.8, 35.4, 34.2, 33.9, 31.2, 28.0, 27.2, 26.8, 20.6, 19.9, 18.7, 17.4, 14.6, 12.5, 12.0. HRMS: m/z [M + H]^+^ for C_50_H_69_N_3_O_10_, calculated 872.5055; observed 872.4967. UPLC purity: 97.30%. t_R_ (HPLC) = 22.32 min. [α]_D_^25^ + 29.412° (*c* 0.0017, CH_3_OH).

### Biology

#### Parasites

Luciferase-expressing *P. berghei* sporozoites^58^ were obtained by dissection of salivary glands of female *Anopheles stephensi* mosquitoes, reared at Instituto de Medicina Molecular.

#### Cell culture

Huh7 cells, a human hepatic cell line was provided by Cenix Bioscience GmbH, and were cultured in complete culture medium, *i.e.* RPMI 1640 supplemented with 10% (v/v) fetal bovine serum, 1% (v/v) glutamine, 1% (v/v) penicillin/streptomycin, 1% non-essential amino acids, and 10 mM HEPES at 37 °C, 5% CO_2_. Cells were plated in 96-well plates at 1 × 10^4^ cells/well 24 h prior to infection.

#### In vitro liver-stage activity assessment

The activity of compounds against *P. berghei*-infected Huh7 cells was assessed in vitro by bioluminescence, as previously described^58^. Briefly, Huh7 cells were seeded as indicated above on the day prior to infection. Compound stock solutions were prepared in DMSO. Test concentrations were obtained by serial dilution of compound stock solutions in infection medium, *i.e.* complete culture medium supplemented with 50 µg/mL of gentamicin and 0.8 µg/mL of fungizone. After 1 h of incubation with selected compound dilutions, 1 × 10^4^ luciferase-expressing *P. berghei* sporozoites were added per well. Plates were centrifuged and incubated for 46 h at 37 °C, 5% CO_2_. At this timepoint, the impact of the compounds on cell viability was assessed by the AlamarBlue (Invitrogen) assay, according to the manufacturer’s recommendations. Next, compounds’ impact on parasite load was assessed by bioluminescence, employing the Firefly Luciferase Assay Kit 2.0 (Biotium).

#### In vitro blood-stage activity assessment

Ring-stage synchronized cultures of *P. falciparum* NF54 at 2.5% hematocrit and at approximately 1% parasitemia were incubated with drugs or DMSO (vehicle control) in 96 well-plates, for 48 h, at 37 °C in a 5% CO_2_ and 5% O_2 _atmosphere. Stock solutions of all compounds were prepared in DMSO. Working solutions were prepared from the stock solutions in complete malaria culture medium (CMCM), which consists of RPMI 1640 supplemented with 25 mM HEPES, 2.4 mM L-glutamine, 50 μg/mL gentamicin, 0.5% w/v Albumax, 11 mM glucose, 1.47 mM hypoxanthine and 37.3 mM NaHCO_3_. For each measurement 5 µl of the culture (approximately 800 000 cells) was stained with the DNA-specific dye SYBR green I at 1 ×. After 20 min of incubation, in the dark, the stained sample was analyzed by flow cytometry using Cyflow Cube 6 (Sysmex, Germany). Approximately 100,000 events were analyzed in each flow cytometry measurement. All samples were analyzed in triplicate and three different experiments were performed.

#### Statistical analyses

Nonlinear regression analysis using GraphPad Prism 8 (GraphPad software, La Jolla California, USA) was employed to fit the normalized results of the dose–response curves for IC_50_ determinations of in vitro hepatic and blood stage activities.

## Supplementary Information

Supplementary Information.

## Abbreviations

CQ: Chloroquine
PQ: Primaquine
ACT: Artemisinin-based combination therapy
WHO: World Health Organization
COSY: Correlation spectroscopy
NOESY: Nuclear overhauser effect spectroscopy
TBDMS: *tert-*Butyldimethylsilyl
CMCM: Complete malaria culture medium
CDI: 1,1'-Carbonyldiimidazole
DMSO: Dimethyl sulfoxide
