# Supplementary material for: Synthesis and antiplasmodial activity of regioisomers and epimers of second-generation dual acting ivermectin hybrids
Source: Sci Rep. 2022 Jan 12;12:564. doi: 10.1038/s41598-021-04532-w (PMC8755717; doi:10.1038/s41598-021-04532-w)
Supplement: Supplementary file 1 — Supplementary Information. [file 41598_2021_4532_MOESM1_ESM.doc]

**Synthesis and Antiplasmodial Activity of Regioisomers and Epimers of Second-Generation Dual Acting Ivermectin Hybrids**

Lovepreet Singh,^1^ Diana Fontinha,^2^ Denise Francisco,^2^ Miguel Prudêncio^2^ and Kamaljit Singh^1^,*

^1^Department of Chemistry, Guru Nanak Dev University, Amritsar – 143 005, India

^2^Instituto de Medicina Molecular, Faculdade de Medicina da Universidade de Lisboa, Av. Prof. Egas Moniz, 1649-028 Lisboa, Portugal

| **Table of Contents** | | |
| --- | --- | --- |
| Sr. No. | Content | Page number |
| 1 | Figure S1. ^1^H and ^13^C NMR spectra of **10**. | 3 |
| 2 | Figure S2. ^1^H and ^13^C NMR spectra of **12a**. | 4 |
| 3 | Figure S3. ^1^H and ^13^C NMR spectra of **12b**. | 5 |
| 4 | Figure S4. ^1^H and ^13^C NMR spectra of **15a**. | 6 |
| 5 | Figure S5. ^1^H and ^13^C NMR spectra of **15b**. | 7 |
| 6 | Figure S6. HRMS Spectrum **10**. | 8 |
| 7 | Figure S7. HRMS Spectrum **12a**. | 8 |
| 8 | Figure S8. HRMS Spectrum **12b**. | 9 |
| 9 | Figure S9. HRMS Spectrum **15a**. | 9 |
| 10 | Figure S10. HRMS Spectrum **15b**. | 10 |
| 11 | Figures S11. FTIR spectrum of **10**. | 10 |
| 12 | Figures S12 and S13. FTIR spectrum of **12a** and **12b**. | 11 |
| 13 | Figures S14 and S15. FTIR spectrum of **15a** and **15b**. | 12 |
| 14 | Figure S16. HPLC chromatogram of **12**. | 13 |
| 15 | Figure S17. UPLC chromatogram of **12a**. | 14 |
| 16 | Figure S18. UPLC chromatogram of **12b**. | 14 |
| 17 | Figure S19. HPLC chromatogram of **15**. | 15 |
| 18 | Figure S20. HPLC chromatogram of **15a**. | 16 |
| 19 | Figure S21. HPLC chromatogram of **15b**. | 17 |
| 20 | Figure S22. UPLC chromatogram of **15a**. | 18 |
| 21 | Figure S23. UPLC chromatogram of **15b**. | 18 |
| 22 | Figure S24. ^1^H-^1^H COSY spectrum of **15a**. | 19 |
| 23 | Figure S25. ^1^H-^1^H COSY spectrum of **15b**. | 20 |
| 24 | Figures S26 and S27. NOESY spectrum of **15a** and **15b**. | 21 |


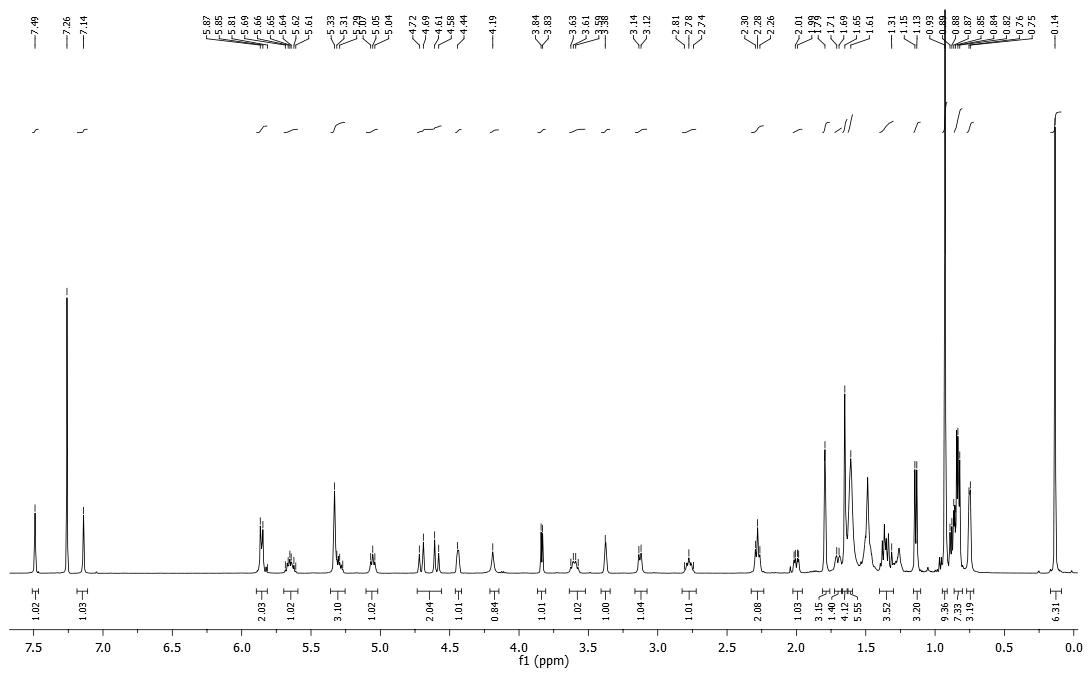

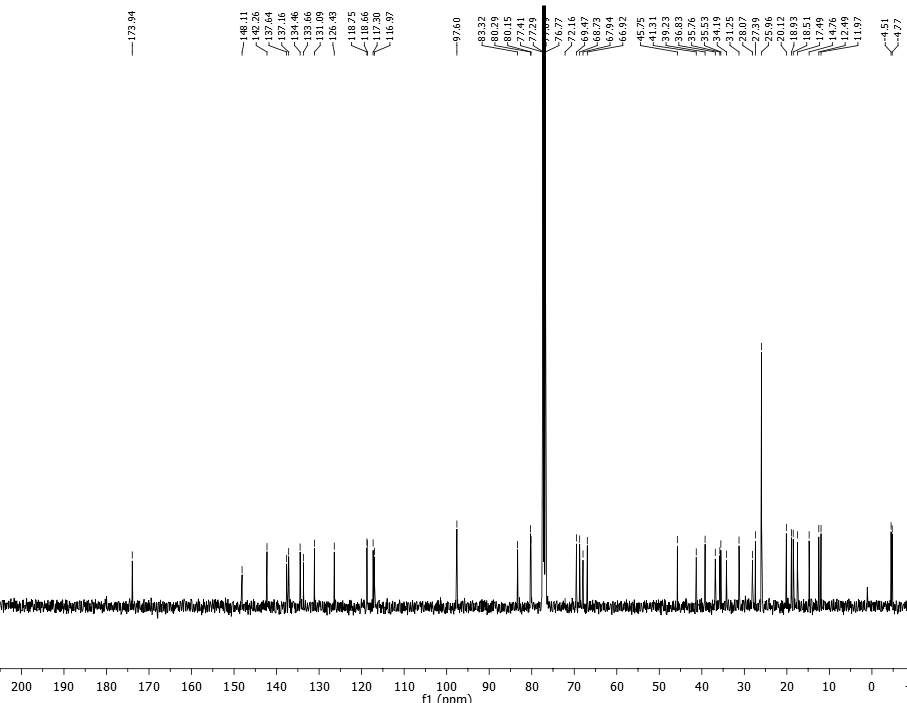


**Figure S1.** ^1^H NMR (500 MHz, CDCl_3_) and ^13^C NMR (125 MHz, CDCl_3_) spectra of **10**.


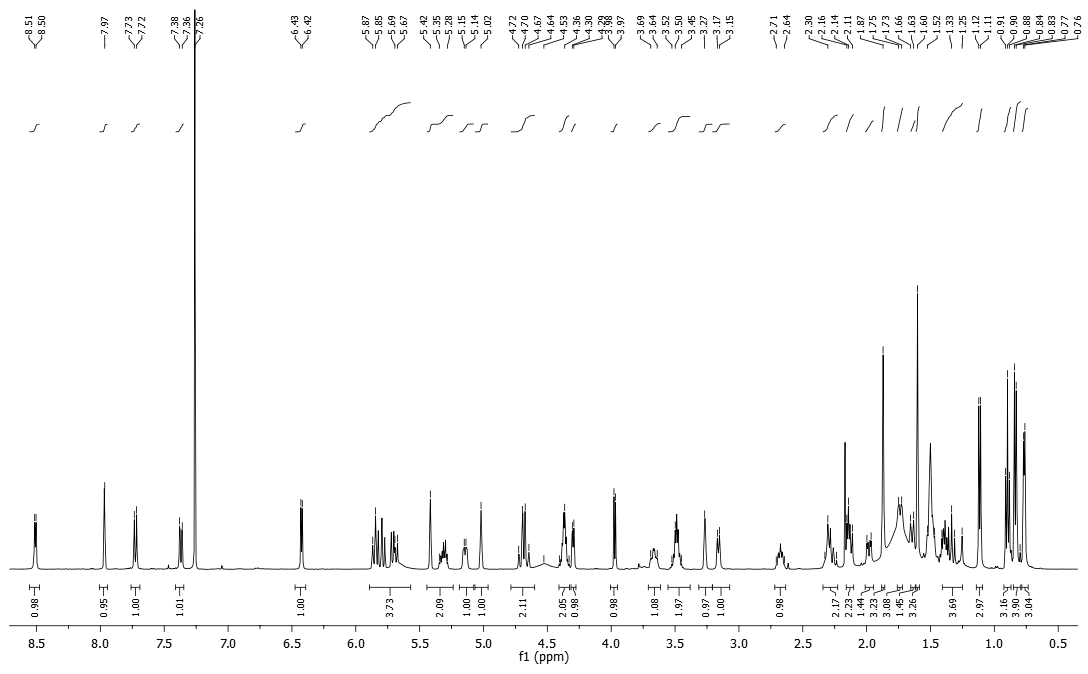

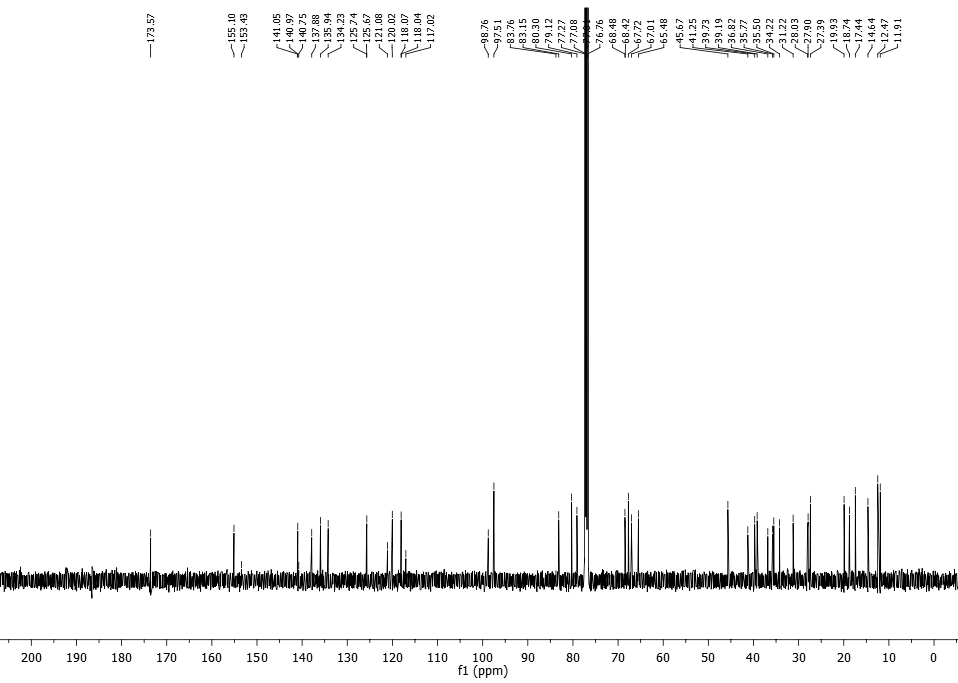


**Figure S2.** ^1^H NMR (500 MHz, CDCl_3_) and ^13^C NMR (125 MHz, CDCl_3_) spectra of **12a**.


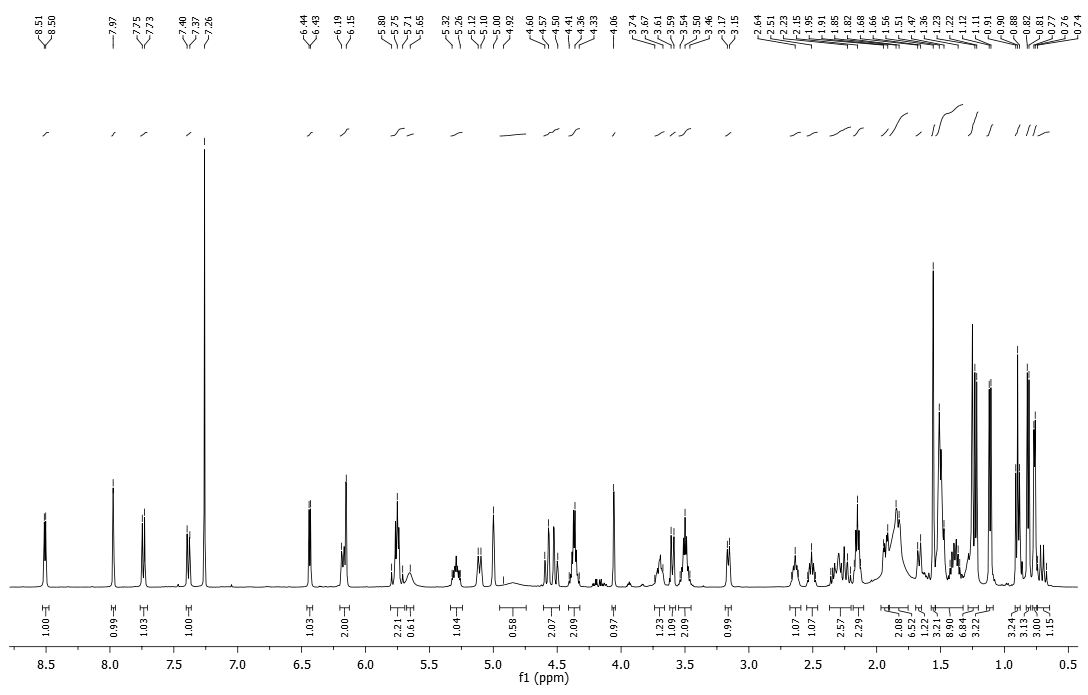

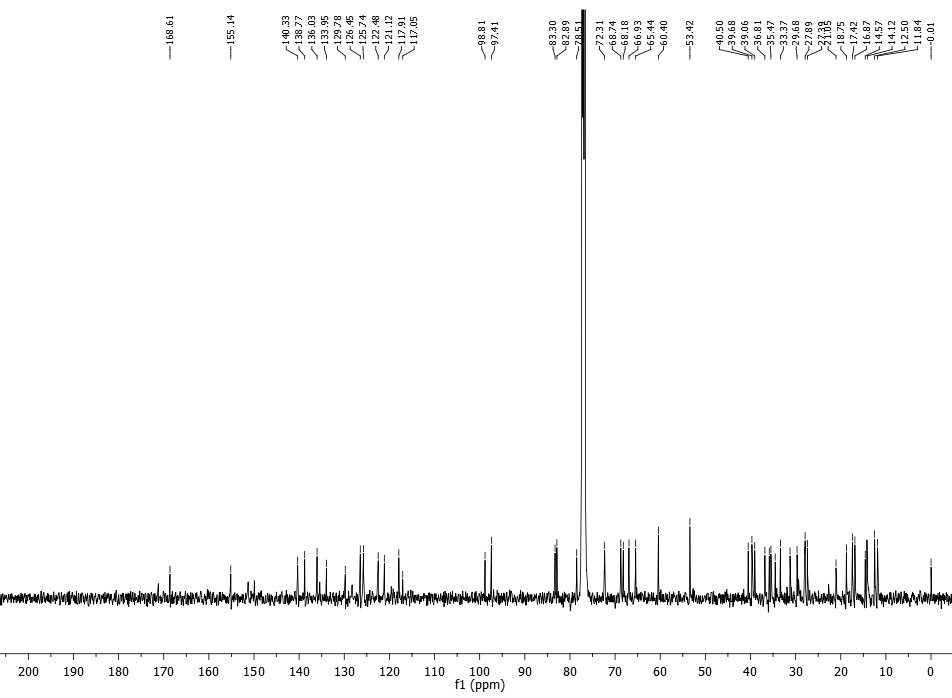


**Figure S3.** ^1^H NMR (500 MHz, CDCl_3_) and ^13^C NMR (125 MHz, CDCl_3_) spectra of **12b**.


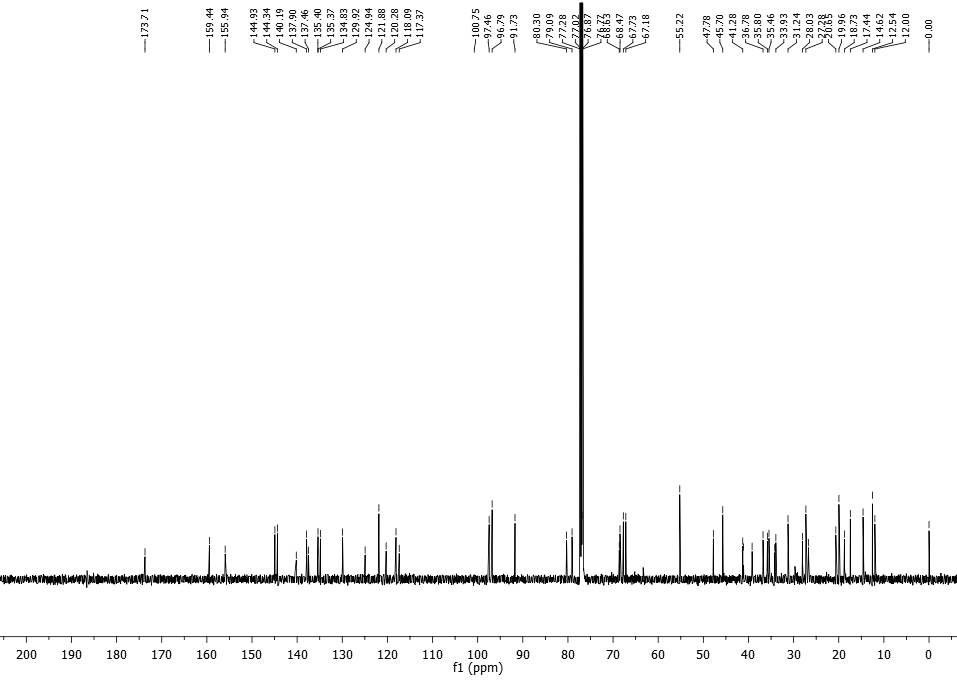

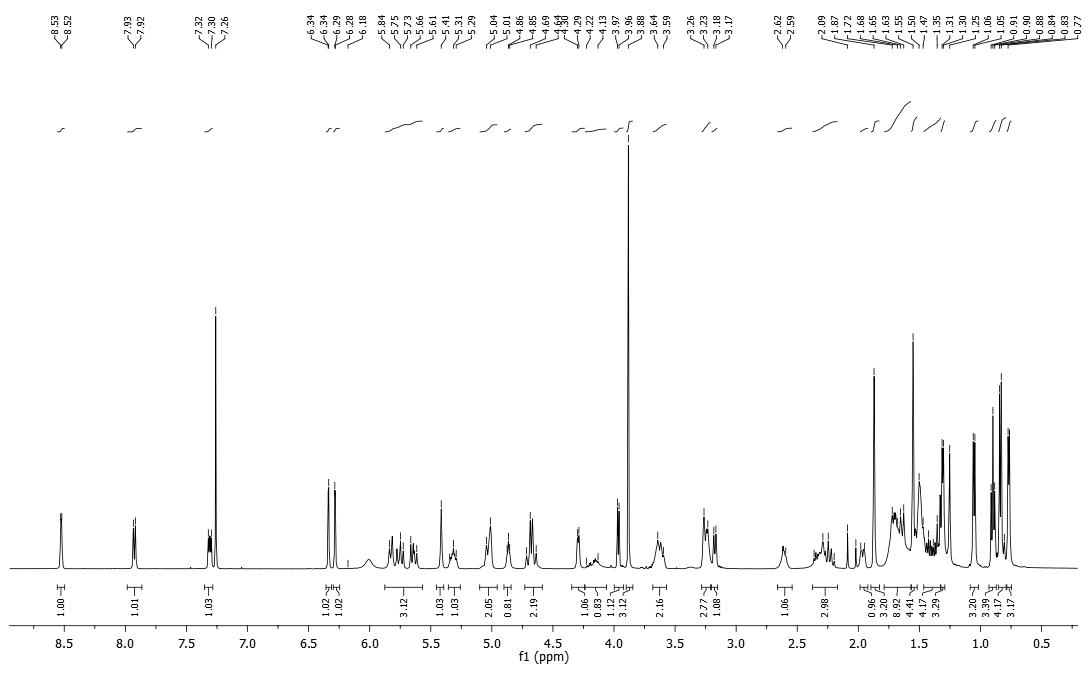


.

**Figure S4.** ^1^H NMR (500 MHz, CDCl_3_) and ^13^C NMR (125 MHz, CDCl_3_) spectra of **15a**.


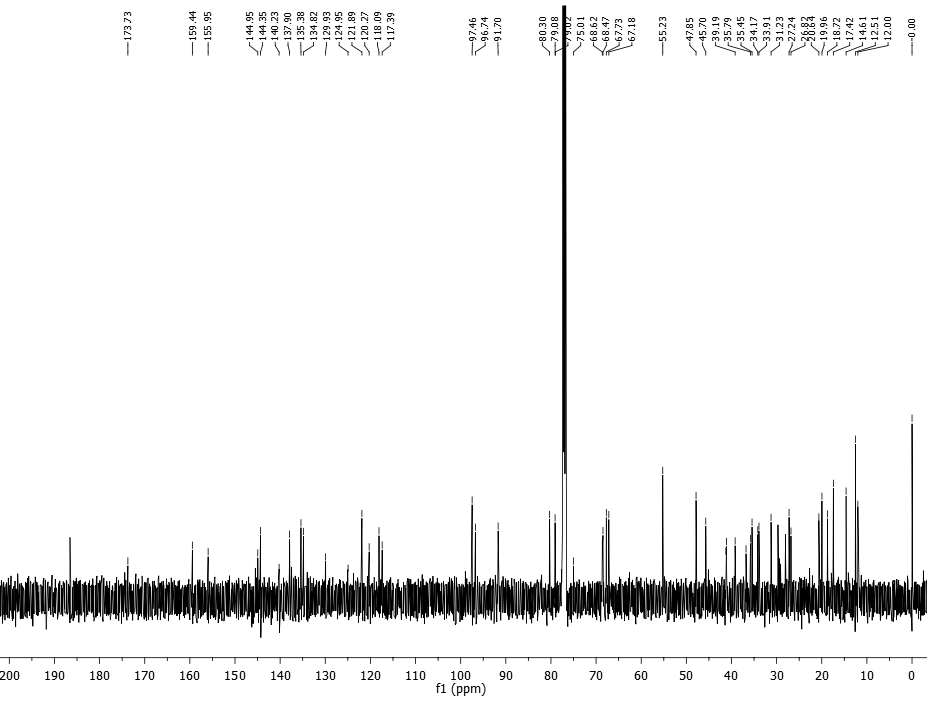

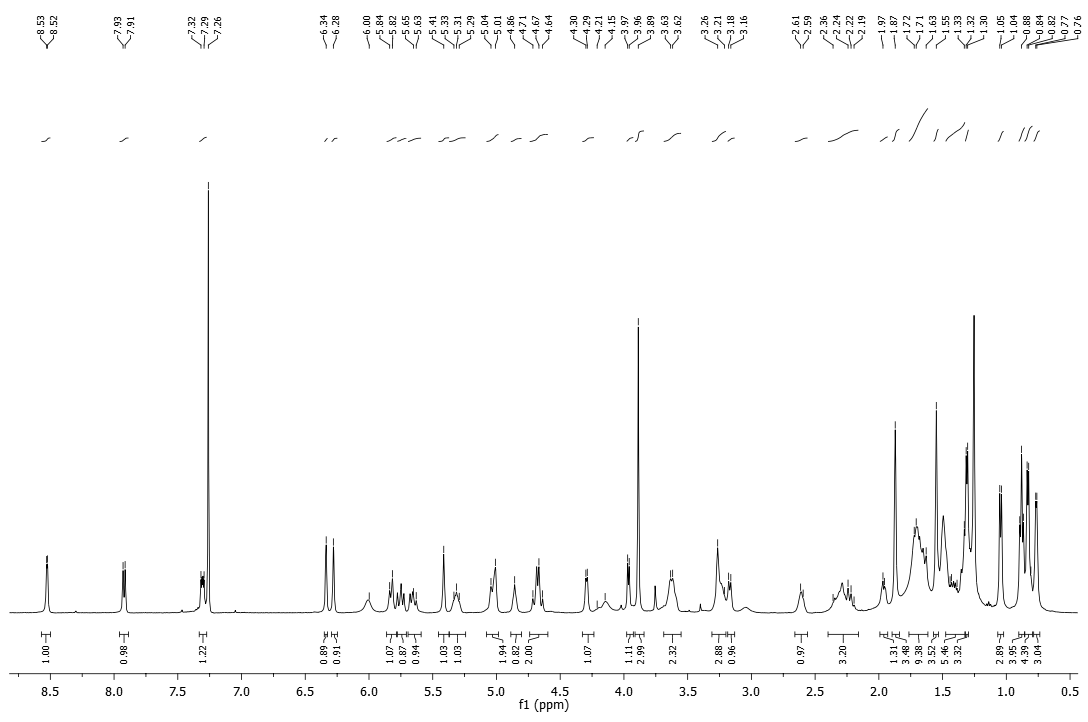


**Figure S5.** ^1^H NMR (500 MHz, CDCl_3_) and ^13^C NMR (125 MHz, CDCl_3_) spectra of **15b**.


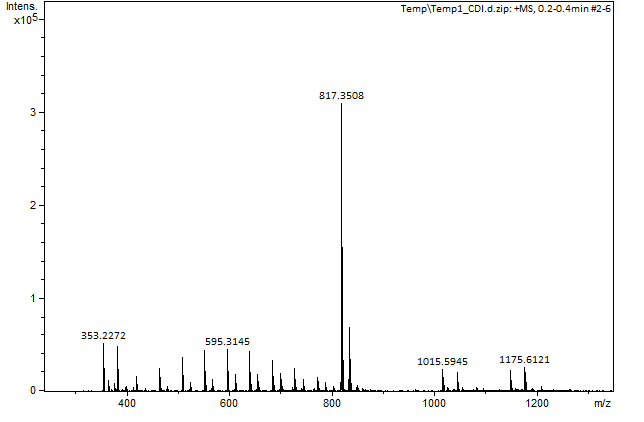


**Figure S6.** HRMS spectrum of **10.**

**
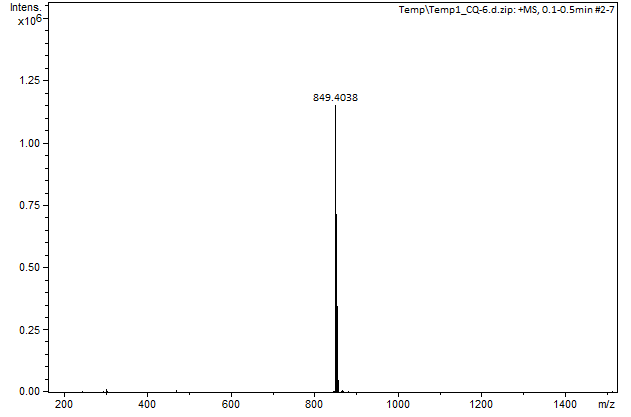
**

**Figure S7.** HRMS spectrum of **12a**.


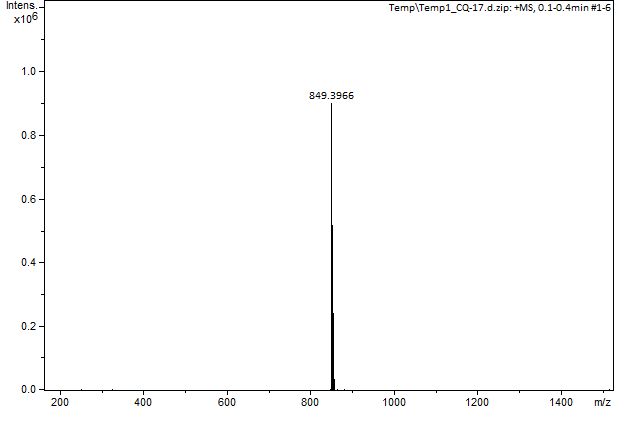


**Figure S8.** HRMS spectrum of **12b**.

**
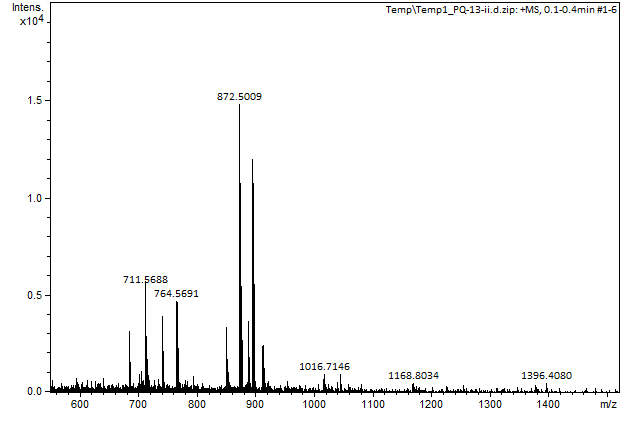
**

.

**Figure S9.** HRMS spectrum of **15a**.


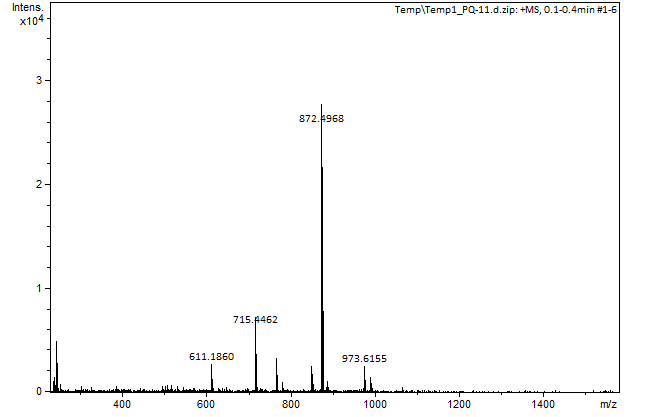


**Figure S10**. HRMS spectrum of **15b**.


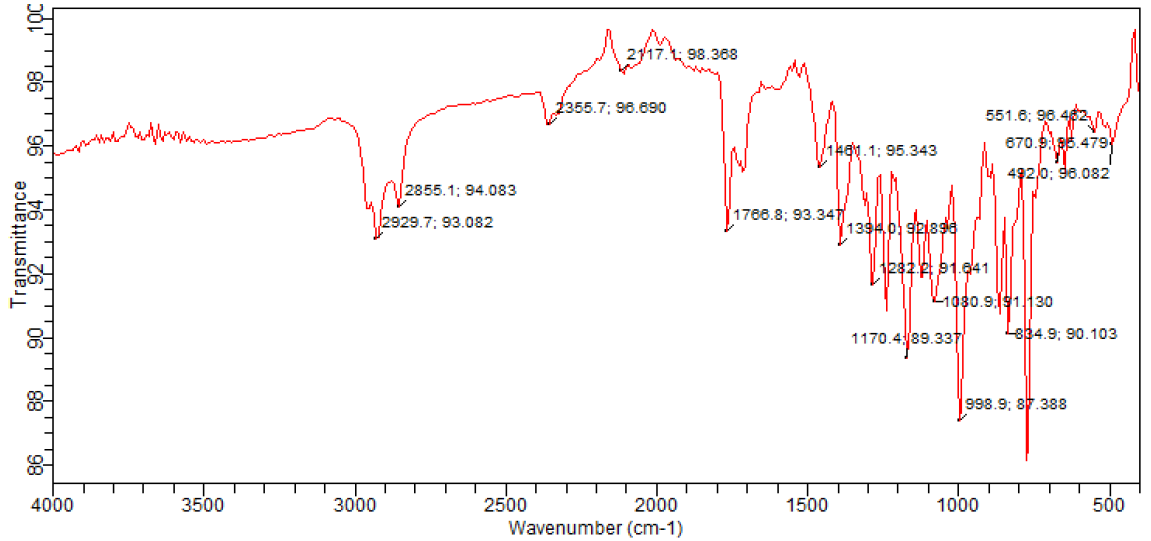


**Figure S11**. FTIR spectrum of **10**.


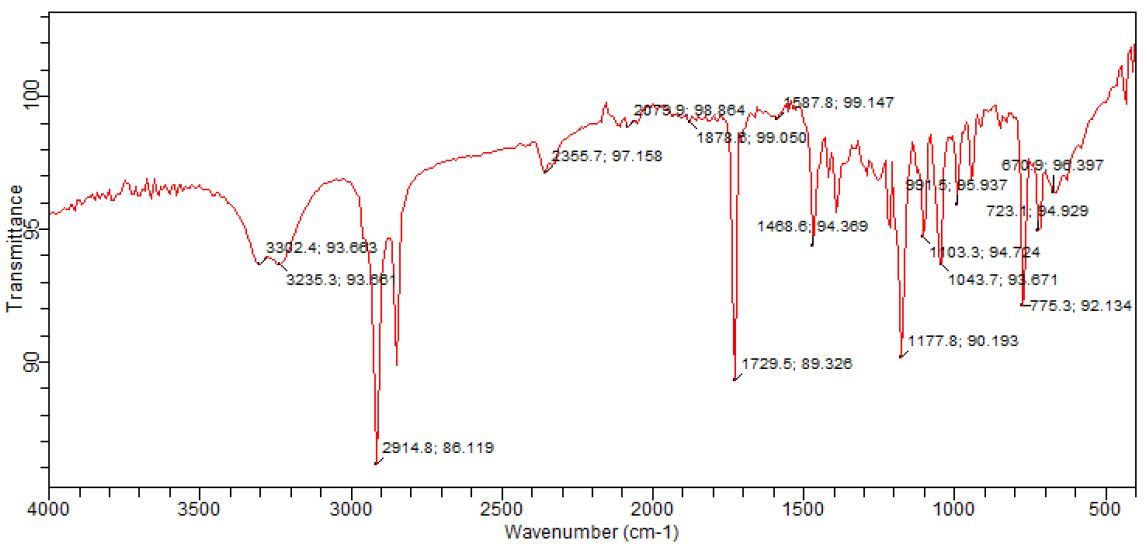


**Figure S12.** FTIR spectrum of **12a**.


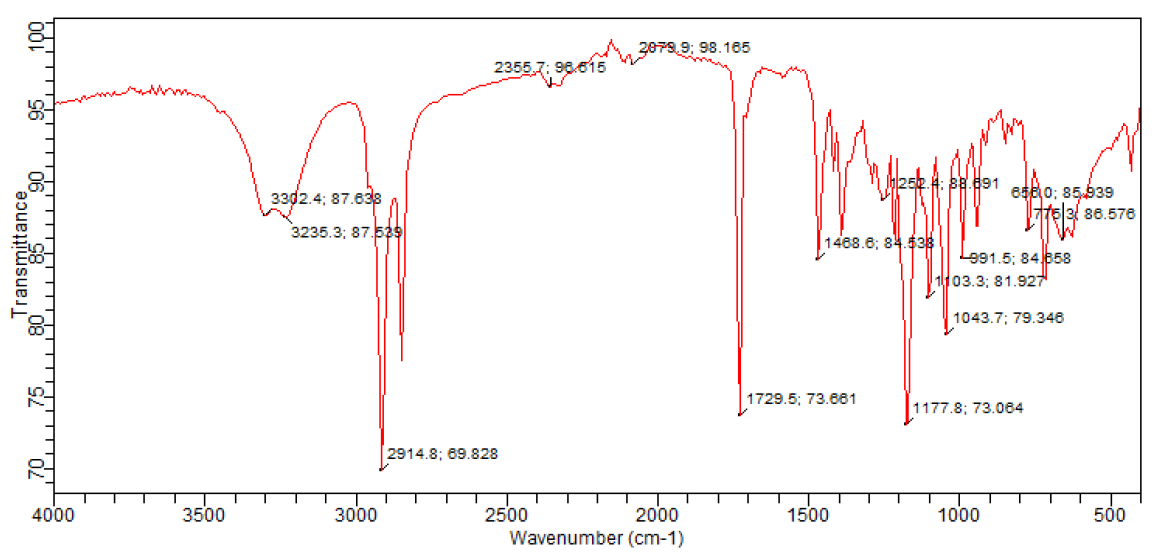


**Figure S13**. FTIR spectrum of **12b**.


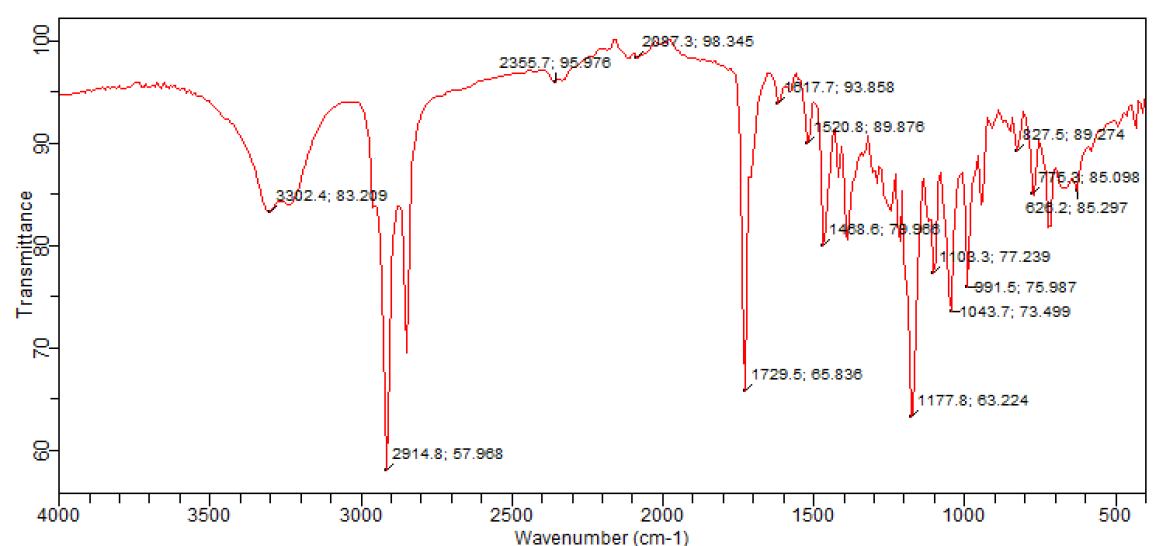


**Figure S14**. FTIR spectrum of **15a**.


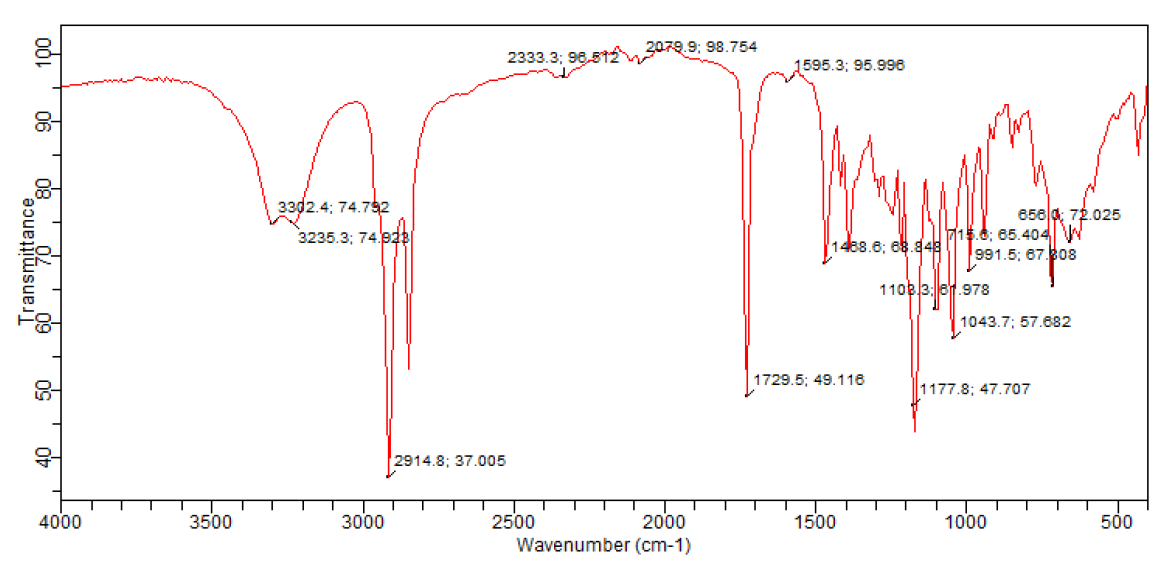


**Figure S15**. FTIR spectrum of **15b**.


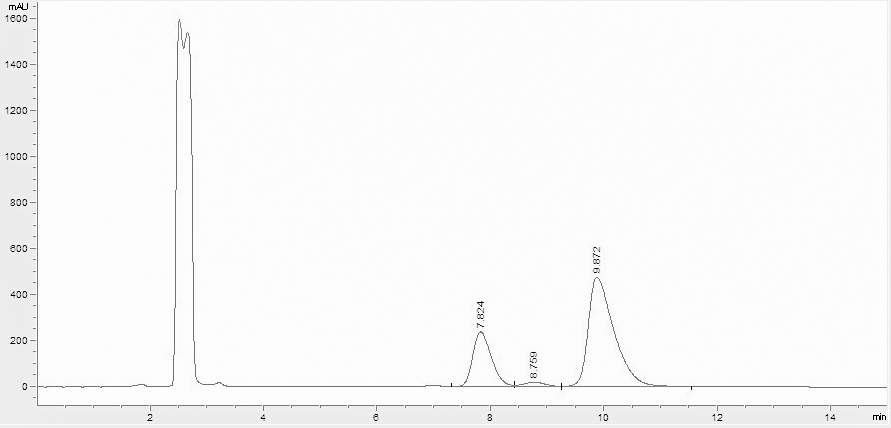


Peak Results

|  | **RT** | **% Area** |
| --- | --- | --- |
| **1** | 7.82 | 25 |
| **2** | 9.87 | 71 |

**Figure S16**. HPLC chromatogram of **12** (**12a + 12b**).

**
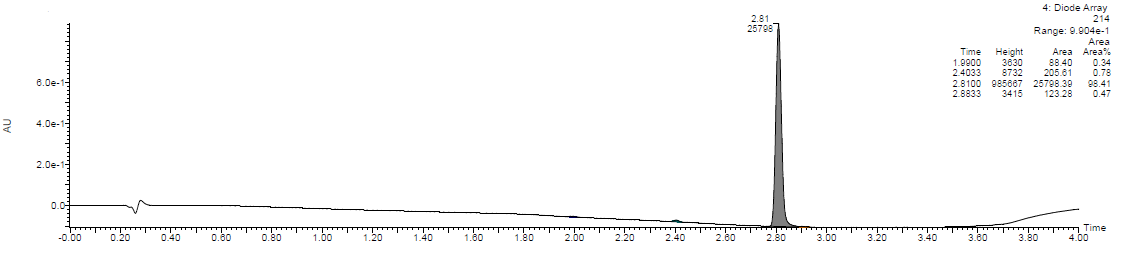
**

**Figure S17**. UPLC chromatogram of **12a**.


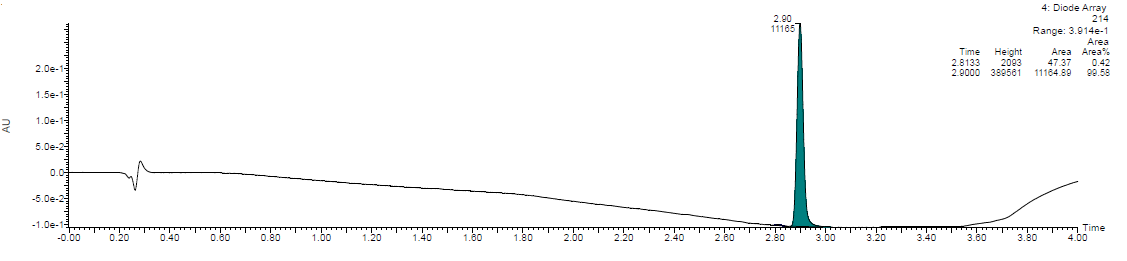


**Figure S18**. UPLC chromatogram of **12b**.

**Figure S19**. HPLC chromatogram of **15** (**15a + 15b**).

**Figure S20**. HPLC chromatogram of **15a**.

**Figure S21**. HPLC chromatogram of **15b**.


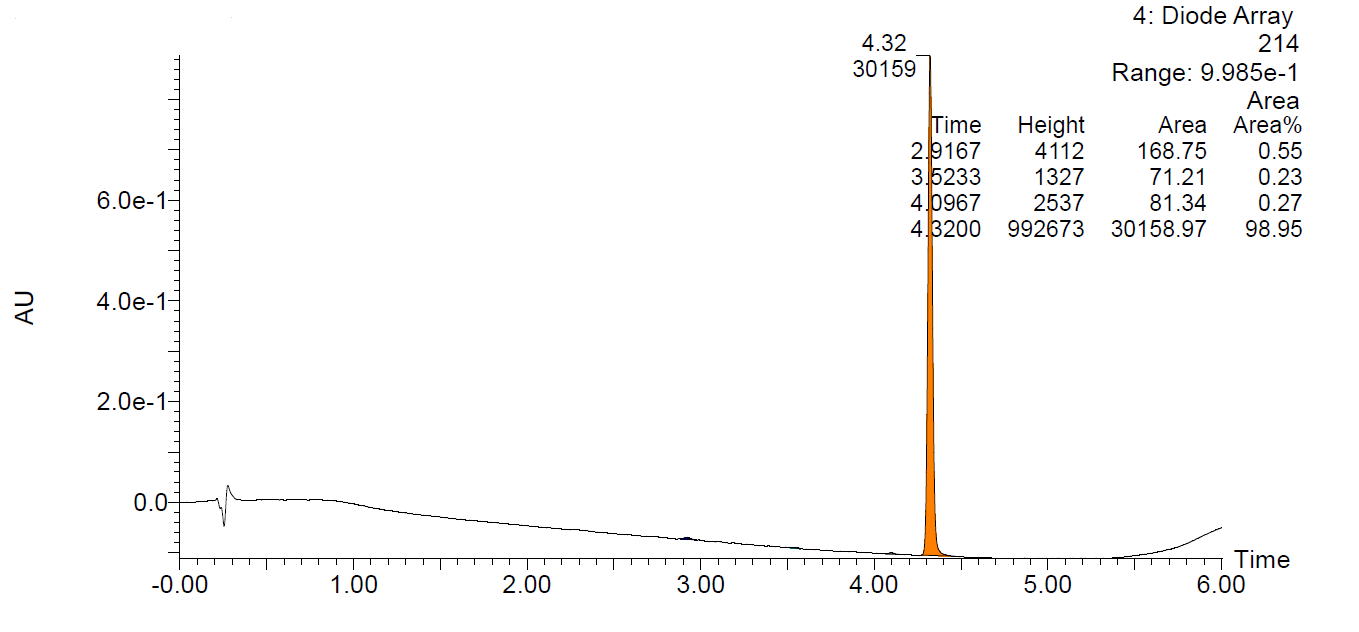
**Figure S22**. UPLC chromatogram of **15a**.


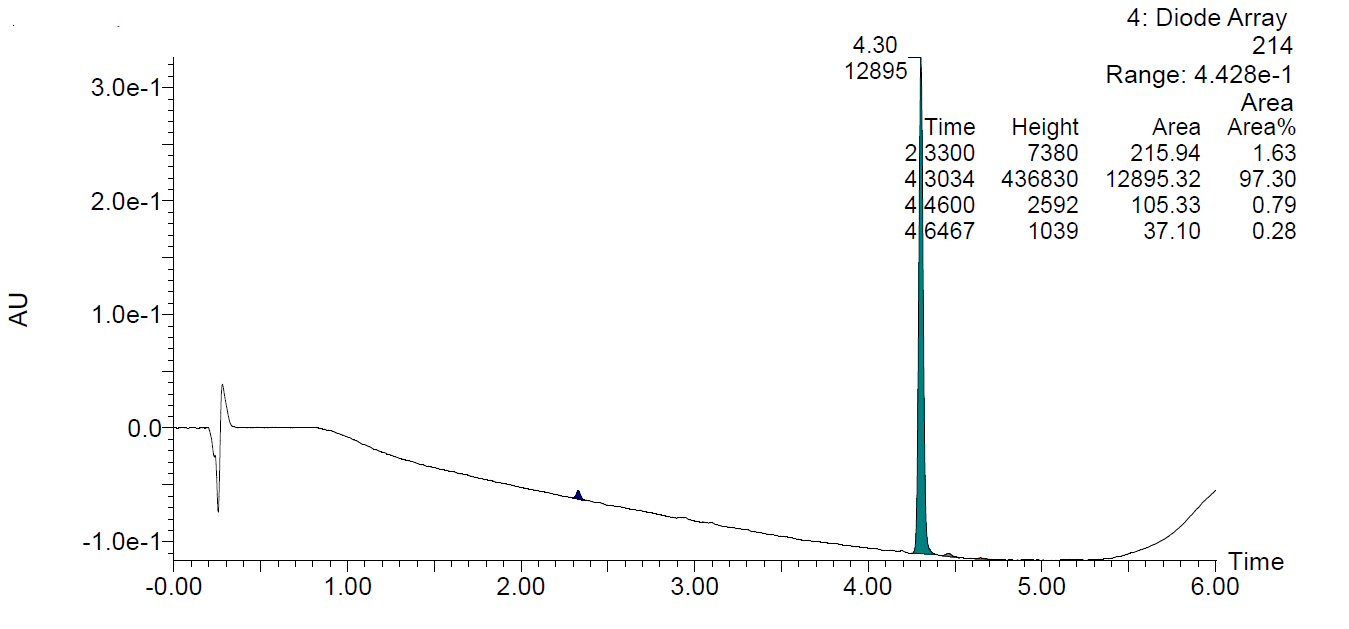


**Figure S23**. UPLC chromatogram of **15b**.


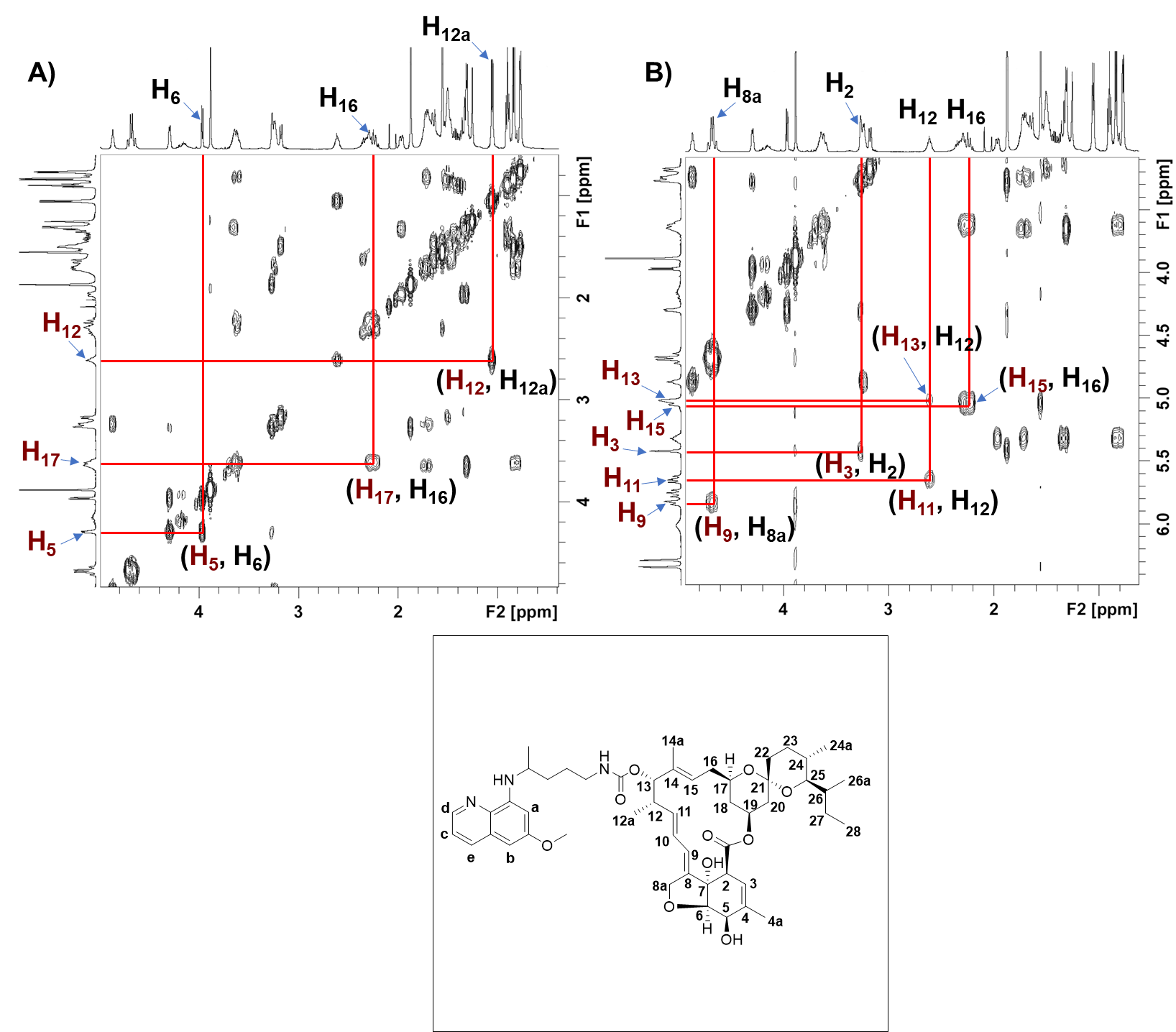


**Figure S24**. ^1^H-^1^H COSY spectrum of **15a**. (The spectrum was viewed in Bruker’s TopSpin 3.6.2 software and corresponding co-relations are highlighted using Microsoft Powerpoint 2019 and the structure was drawn using ChemDraw 20.0).


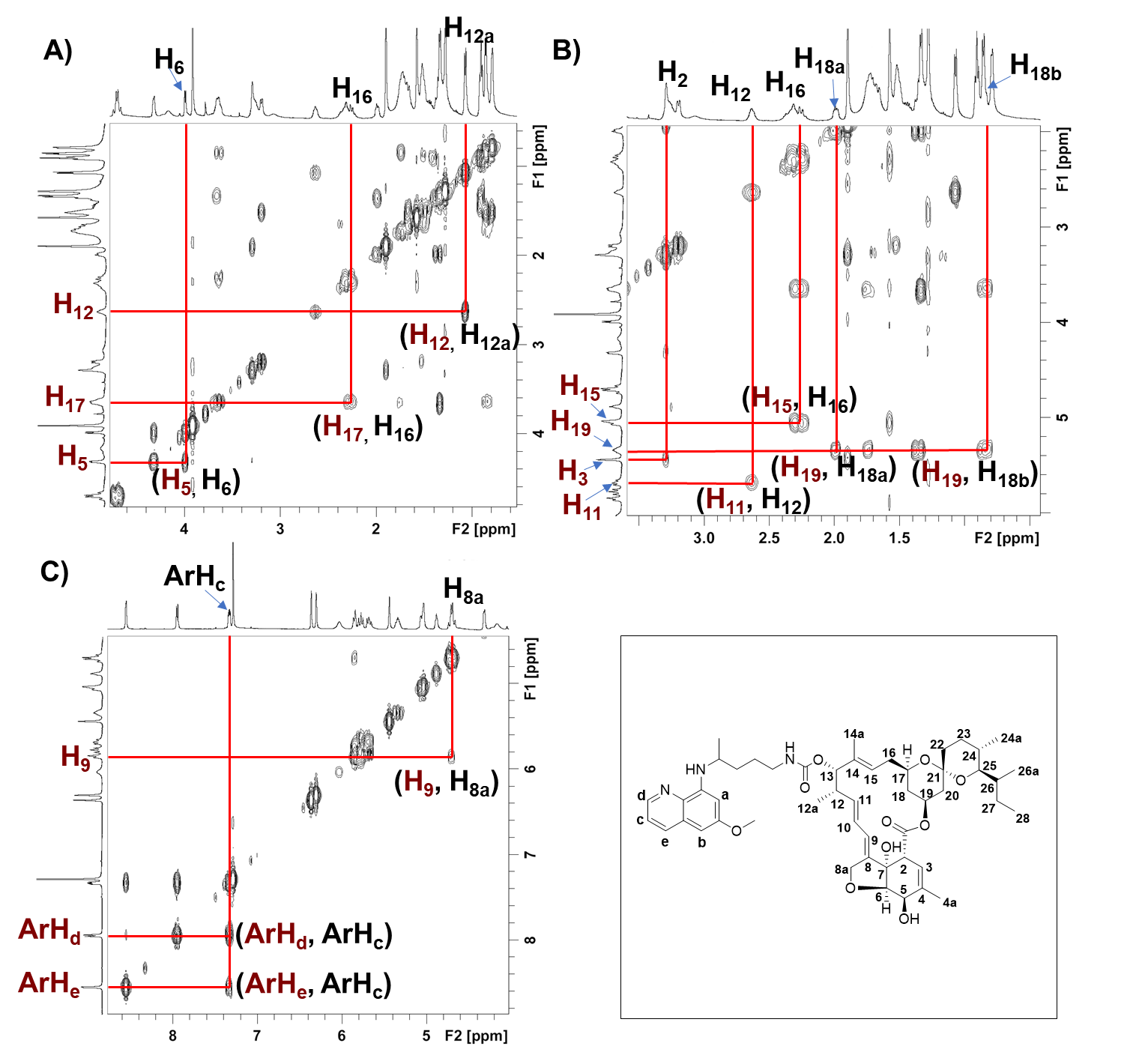


**Figure S25**. ^1^H-^1^H COSY spectrum of **15b**. . (The spectrum was viewed in Bruker’s TopSpin 3.6.2 software and corresponding co-relations are highlighted using Microsoft Powerpoint 2019 and the structure was drawn using ChemDraw 20.0).

**Figure 26**. NOESY spectrum of **15a**. (The spectrum was viewed in Bruker’s TopSpin 3.6.2 software and corresponding co-relations are highlighted using Microsoft Powerpoint 2019).

**Figure 27**. NOESY spectrum of **15b**. . (The spectrum was viewed in Bruker’s TopSpin 3.6.2 software and corresponding co-relations are highlighted using Microsoft Powerpoint 2019).
